# Macromolecules in polysorbate 80 for injection: an important cause of anaphylactoid reactions

**DOI:** 10.1186/s40360-022-00591-5

**Published:** 2022-07-18

**Authors:** Yue Li, Jinlian Duan, Heng Xia, Yongkun Li, Bin Shu, Weigang Duan

**Affiliations:** 1 School of Basic Medicine, Yunnan University of Traditional Chinese Medicine, 1076, Yuhua Rd., Kunming, 650500 China; 2 School of Traditional Chinese Medicine, Yunnan University of Traditional Chinese Medicine, 1076, Yuhua Rd., Kunming, 650500 China; 3 Jiangsu Center for Safety Evaluation of Drugs, Nanjing Tech University, 30, South Puzhu Rd., Nanjing, 211899 China

**Keywords:** Polysorbate 80 for injection, Anaphylactoid reactions, Macromolecular impurities, Capillary permeability, Passive cutaneous anaphylactoid reaction

## Abstract

Polysorbate 80 for injection (TW80) is a common excipient used for injection whose macromolecular impurities, including those that cause anaphylactoid reactions, are frequently ignored. The main aim of this study was to prove that the macromolecular impurities in the excipient are an important cause of anaphylactoid reactions. Component A (containing macromolecules > 100 kDa), Component B (containing macromolecules from 10 to 100 kDa), and Component C (containing substances < 10 kDa) were prepaired from the original TW80 using ultrafilters. The original TW80 contained numerous substances with molecular weights > 10kD. The original TW80 and Components A and B caused strong anaphylactoid reactions in both guinea pigs and rabbits by intravenous administration. Moreover, the original TW80 and Components A and B even caused strong passive cutaneous anaphylactoid (PCA) reactions and pulmonary capillary permeability. The PCA reaction and increased permeability were partly prevented by cromolyn sodium. Additionally, the original TW80 and Components A and B caused vasodilation and severe hemolysis in vitro. The anaphylactoid reactions were associated with histamine release but not with mast cell degranulation. Nevertheless, Component C almost caused no anaphylactoid reactions or hemolysis and was weaker in the few reactions that ocurred. Taken together, these results suggest that macromolecular substances are one of the main risk factors responsible for anaphylactoid reactions and hemolysis caused by TW80.

**Supplementary Information:**

The online version contains supplementary material available at 10.1186/s40360-022-00591-5.

## Introduction

Polysorbate 80, also named Tween 80, is a commonly used solubilizer and considered as a safe excipient [1]. The excipient is used worldwide in the food, cosmetic, and pharmaceutical industries. The excipient has been approved for injection by authorities in many countries, including China, the United States, and the United Kingdom.

With a higher quality standard, polysorbate 80 for injection (TW80) is a popular excipient used in the modern injection industry [2]. Indeed, drugs such as taxol injection [3] (an antineoplastic agent) and amiodarone injection [4] (an antiarrhythmic agent) contain the excipient. In China, polysorbate 80 for injection was abused in traditional Chinese medicine injections (TCMIs) [2], such as Yuxingcao injection [5], which resulted in a series of nonspecific adverse reactions. As the adverse reactions of the injections with the excipient often happen more frequently and more seriously than those of oral drugs with similar formulae, the added excipient has been suggested as one of the main risk factors [6–8]. Based on clinical observation, most adverse reactions are anaphylactoid reactions rather than anaphylactic reactions because the symptoms can be frequently ignited by the first exposure without activating the patients’ adaptive immune system.

Anaphylactoid reaction is also known as non-allergic reaction, the symptoms of which are very similar to those of anaphylactic (allergic) reaction. It is certain that anaphylactic reaction is mediated by immunoglobin E (IgE) and the symptoms will not appear at the first exposure. Different from anaphylactic reaction, the mechanisms of anaphylactoid reaction may associate the activation of innate immune system, including complements [8], Toll-like receptors (TLRs) [9], and some G protein coupled receptors (MRgprX2) [10]. In addition, the symptoms of anaphylactoid reaction caused by an injection are different from those caused by an oral drug with the same formula [11]. Thus, it can be inferred that the reactions are likely caused by macromolecular impurities from the raw materials, contaminated substances, and excipients, which can be prevented by the gastrointestinal barrier [12].

TW80 is a viscous liquid, which is almost colorless or light yellow. Theoretically, 1 mol of polysorbate 80 can be synthesized from 1 mol of sorbitol, 20 mol of ethylene oxide, and 1 mol of oleic acid [13]. The excipient is synthesized in a three-step process, and the product is a mixture of polymers with a theoretical molecular weight of 1310 Da. Theoretically, the remaining substrates and the substances caused by unwanted polymerization are the impurities. The remaining substrates are strictly controlled by the authorities; however, the unwanted polymers, usually with bigger molecular weights and not conforming to the general formula, should also be considered as impurities as they play a minor role in the drug action. Macromolecular impurities are considered to represent a major contributor to the nonspecific adverse reactions of injections, but are largely ignored by the modern pharmaceutical industry.

In this study, we attempted to isolate the macromolecules of TW80 and verify the risk of anaphylactoid reaction.

## Materials and methods

### Materials

Polysorbate 80 for injection (TW80) (Lot: 20,170,501) was manufactured by Nanjing Well Pharmaceutical Co., Ltd. (Nanjing, China) and was approved by the National Medical Products Administration of China (NMPA). C48/80 was manufactured by Sigma-Aldrich. Normal saline for injection (NS) and water for injection were manufactured by Chenxin Pharmaceutical Co., Ltd. (Jining, China). Enzyme-linked immunosorbent assay (ELISA) kits for Guinea pig IgE (Lot. MM-034001), C-reactive protein (CRP) (Lot. MM-3531202), high mobility group protein (HMGB1) (Lot. MM-8750102), and complement 5a (C5a) (Lot. MM-8750302) were manufactured by Jiangsu Enzyme Industry Co., Ltd. (Yancheng, China). ELISA kits for rabbit soluble complement terminal complex Sc5b-9 (Sc5b-9) (Lot. MM-8253002), complement factor B (CFb) (Lot. MM-8258102), mannose binding lectin (MBL) (Lot. MM-8258801), bradykinin (BK) (Lot. MM-8258201), β-hexosaminidase (β-HEX) (Lot. MM-8257201), lysozyme (LZM) (Lot. MM-8258601), and histamine (HIS) (Lot. MM-023801) were manufactured by Jiangsu Enzyme industry Co., Ltd. (Yancheng, China). Cromolyn sodium (Lot. D16J12G137501) and Evans Blue (Lot. L04D11J133367) were manufactured by Shanghai Yuanye Biotechnology Co., Ltd. (Shanghai, China). Histamine phosphate of the analytic impurity product was manufactured by Beijing Bo’aotuo Technology Co., Ltd (Beijing, China). Hematoxylin–eosin (HE) staining kits were manufactured by Boster Biological Technology Co., Ltd. (Wuhan, China).

Non-pregnant guinea pigs weighing 250–350 g, female Sprague–Dawley (SD) rats weighing 180–220 g, rabbits weighing 2.5–3.5 kg, and a male cat weighing 3 kg were provided by Kunming Medical University. The animals were housed at 22 °C temperature, at 45–55% humidity-controlled conditions, and under natural light. At the end of the animal experiments, the animals were intraperitoneally anesthetized with urethane (1.0 g/kg). Under deep anesthesia, the necks of the animals were dislocated for euthanasia. The bodies of the animals were collected in yellow plastic bags and kept in a refrigerator at − 20˚C until they were taken away by a green company for cremation.

The KA6600A multiple microplate reader was manufactured by Beijing Kai’ao Technology Development Co., Ltd. (Beijing, China). The FDU-1100 lyophilizer was manufactured by EYELA (Tokyo, Japan). Ultrafilters of 100 kDa (Lot: 1806047VS) and 10 kDa (1808013VS) were manufactured by Sartorius AG (Hamburg, Germany). The biological signal acquisition system (MD3000) was manufactured by Anhui Zhenghua Biological Instrument Equipment Co., Ltd (Huaibei, China); ultrapure water was obtained using a Milli-Q water purification system (EMD Millipore Group, Darmstadt, Germany). The other instruments and reagents used in this study were made in China unless otherwise stated.

### Ethical approval and consent to participate

The animal experiments were approved by the Animal Care and Use Committee of Yunnan Provincial Key Laboratory of Molecular Biology for Sinomedicine, Kunming, China (2018FF001-002), and animal experiments were conducted under the Guidelines for the Ethical Review of Laboratory Animal Welfare of China (GB/T 35,892–2018). Animal were treated in accordance with ARRIVE guidelines (https://arriveguidelines.org).

### Macromolecular substance preparation

The original TW80 was dissolved in water for injection to obtain a 5% solution. The light yellow solution was gradually added to the upper tube of the ultrafilter of 100 kDa. The ultrafilter was spun at 3,500 g and 4 °C until more than a half volume was filtered through. The solution that had passed through the filter was collected, which contained substances with molecular weights < 100 kDa, and the volume in the upper tube was recovered by mixing with the water for injection and spun again. When the solution in the upper tube was diluted six times and ultrafiltered, the detained solution in the upper tube was collected as Component A, which contained substances with molecular weights > 100 kDa. Similarly, the solution passed through the 100-kDa ultrafilter was transferred to another upper tube of 10 kDa and spun again. The solution that had filtered through was collected as Component C (< 10 kDa), while the retained part was Component B (from 10 to100 kDa).

The abovementioned three components were dried by a lyophilizer. Briefly, the solution was transferred to a plastic bottle of 250 ml, and frozen at –40 oC overnight. The frozen solution was put into the lyophilizer chamber and lyophilized for > 48 h. Subsequently, the bottle was weighed every 3 h, and when the total weight of the bottle decreased no further, the lyophilization was ended. The net weight of the oily substances was calculated by subtracting the bottle weight from the total weight.

### Systemic anaphylactic and anaphylactoid reaction in guinea pigs evoked by macromolecules

The original TW80 and its components were dissolved in NS (100 mg/ml). Every six guinea pigs, including three male and three female animals (non-pregnant) were randomly arranged in a group to detect anaphylactic or anaphylactoid reactions. Animals administered NS in the same volume served as a negative control. The detection of the anaphylactic reaction was performed according to the Chinese Pharmacopoeia (CP) of the 2015 edition (Book 4) [14]. Briefly, a solution of 0.5 ml (15 mg/ml) was intraperitoneally administered every 2 days for three doses, and an injection of the same volume was intravenously administered 2 weeks later to evoke the anaphylactic reaction. The symptoms appeared 30 min after the evocation was observed, and their blood was drawn. The animals continued to be observed for 7 days, and their blood was drawn on the last day. Serum was collected from the collected blood, and factors in the serum were assayed (see below).

For the anaphylactoid reaction test, each guinea pig was intravenously administered with original TW80 and its components (15 mg, 1 ml). Animals administered with NS of the same volume served as the negative control. The symptoms appeared 30 min after administration, following which, blood was immediately drawn without anticoagulation to obtain serum samples.

Scores were recorded based on the symptoms (Table 1). If an animal exhibited two or more symptoms, only the higher or the highest score was used [11].

**Table 1:** Scores of symptoms

Score	Symptom	Score	symptom
1	Piloerection	8	dyspnea
2	Tremble	9	gatism
3	frequent scratching nose	10	unsteady walking or falls
4	sneezing three times in a row	11	spasm or convulsion
5	Three coughs in a row	12	Shock
6	Retching	13	death
7	Cyanosis		

The coagulated blood was centrifuged at 3,000 g and 4 °C for 5 min to obtain serum. The levels of IgE, HIS, CRP, HMGB1 and C5a in the serum were determined using ELISA kits according to the protocols provided by the manufacturer.

Systemic anaphylactoid reaction in rabbits evoked by macromolecules.

The original TW80 and its components were dissolved in NS (100.0 g/L). Every six rabbits, including three male and three female animals (non-pregnant) were randomly arranged in a group to detect anaphylactoid reactions. Animals administered NS of the same volume served as the negative control. Briefly, the solution (70 mg, 0.70 ml) was intraperitoneally administered. The symptoms appeared 30 min after the evocation was observed, following which, blood was drawn to obtain serum for the assay.

The scores were recorded according to Table 1. If an animal exhibited two or more symptoms, only the higher or the highest score was used. The levels of SC5b-9, CFb, MBL, BK, β-HEX, LZM, and HIS in the serum were determined using ELISA kits according to the manufacturer’s protocols.

### Substances released from leukocytes evoked by macromolecules

Guinea pigs were anesthetized with urethane (1.0 g/kg) by intraperitoneal injection. Next, their abdomens were opened and their anticoagulated blood using heparin was drawn via their abdominal aortae. The blood was spun at 1,000 g and 4 °C for 5 min to remove erythrocytes, and the volume was recovered by adding NS. The suspensions from different guinea pigs were mixed, and a 5 ml suspension containing whole white leukocytes was transferred to the wells of a six-well plate. When the original TW80 or its components of 16 μg (10%, 160 μl) were added to the suspension and mixed, the plate was kept at 37 °C for 30 min. C48/80 (20 μg/ml) served as the positive control. A 1 ml suspension was sampled and centrifuged at 1,000 g and 4 °C for 5 min to obtain the supernatant. The levels of β-HEX, LZM and HIS in the supernatant were determined using ELISA kits.

### Passive cutaneous anaphylactoid (PCA) reaction evoked by macromolecules in guinea pigs

Every four guinea pigs, including two male and two female (non-pregnant) weighing approximately 300 g, were randomly arranged in a group. The animals were pretreated with cromolyn sodium (50 mg/kg) or NS of the same volume by intraperitoneal injection for 5 days. On the day before the administration, the back fur was removed with a pair of scissors. On the second day, they were intravenously injected with 1 ml Evans Blue solution (0.5%, dissolved in NS). Fifteen minutes later, the original TW80 and its components (10%; 0.15 ml) were subcutaneously injected at one point. The administration of NS of the same volume served as the negative control, and the administration of C48/80 (0.50 mg/ml, 0.15 ml) served as the positive control. Thirty minutes later, the animals were anesthetized with urethane (1.0 g/kg), their back skin was cut, and the blue-staining areas were photographed. The Evans Blue in the skin (4.00 cm^2^) was extracted using 4 ml acetone-NS solution (acetone: NS = 7:3) for 24 h. The extract was centrifuged at 3,000 g for 5 min, and the intensity of Evans Blue in the supernatant was determined by a multiplate reader at 610 nm.

Following the extraction of Evans Blue, a piece of skin around the injection point was selected. The skin was fixed with the acetone solution routinely dehydrated with ethanol, and embedded with paraffin. The skin was cut at 5 μm and stained with HE staining kits. The sections were scanned using a fluorescence microscope in a light mode.

### Pulmonary vascular permeability affected by macromolecules

Female rats were intraperitoneally treated with or without 10 mg cromolyn sodium. Thirty minutes later, the rats were anesthetized with urethane (1 g/kg). Next, their abdomens and chests were opened, and their superior and inferior venae cava were ligated with artery clamps. The NS was perfused into the right ventricle under a pressure of 1,500 mmH_2_O. Their abdominal aortae were cut for discharge. When the pulmonary circulation was perfused for 5 min, the color of the lungs became light, and the almost-colorless perfusate discharged from their aortae. Then, the original TW80 or its components (11.25 mg/ml; 1 ml) were perfused into the system and maintained for 10 min. Subsequently, Evans Blue solution (50 mg in 1000 mL NS; 1 ml) was perfused into the system and maintained for 10 min. Finally, the system was perfused with NS for 5 min, and the lungs were removed from their chests and photographed. The presence of blue staining in the lungs suggested vascular permeability.

### Blood pressure affected by macromolecules

The cat was anesthetized with urethane (1.0 g/kg) and fixed on an operating table, with the local ambient temperature maintained at 30 oC. The skin of the cat’s neck was cut to expose its trachea. After the trachea was linked to a tube to maintain steady breathing, the right common carotid artery was separated and its distal side was ligated. The end of the arterial catheter filled with NS was connected to a blood pressure sensor, and the other end was inserted into the common carotid artery. Blood pressure signals were recorded when the operation was completed. NS, histamine phosphate (0.15 μg/kg, 0.3 ml), or different components of TW80 in the same dose (19.5 mg/kg, 0.3 ml) were successively injected through the cat’s femoral vein. Note that the next injection was only allowed when the blood pressure had recovered.

### Hemolysis affected by macromolecules

To measure the ability of the macromolecules to affect hemolysis, 5 ml of blood was drawn from a rabbit and decanted into a 100-ml glass beaker. The blood was stirred with a glass stick to remove fibrinogen and fibrous protein and obtain blood without fibrinogen. The blood was mixed with 45 ml NS at 10 °C, and centrifuged at 1,000 g and 10 °C for 15 min to rinse the blood. The blood was rinsed with NS thrice, and clear blood was obtained. Next, the clear blood from the original 5-ml blood sample was mixed with 120 ml NS to obtain the blood suspension that was used for the hemolysis assay. The blood suspension (2.5 ml and NS of 2.2 ml) was transferred to a tube, and a solution of the original TW80 and different concentrations of its components (0.3 ml) was added and gently mixed. Meanwhile, the 2.5-ml blood suspension was mixed with 2.5 ml of water for complete hemolysis. The tubes were kept at 37 °C for 3 h, and centrifuged at 1,000 g for 15 min. The absorbance of the supernatant at 610 nm was read by the microplate reader. The hemolysis rate was calculated using the following formula (1).

1 $$Hemolysis \, rate = \frac{{Absorbance_{sample} }}{{Absorbance_{complete \, hemolysis} }} \times 100\%$$

### Statistics

The values are expressed as the mean + standard deviation (SD) or median + inter-quartile range (IQR). If the data exhibited a normal distribution, a one-way analysis of variance (ANOVA) was performed to compare the means between groups. If there was a significance between groups, a post-hoc test (equal variance) or Tamhane’s test (unequal variances) was used to compare the means between the two groups. If the data did not exhibit the normal distribution, a non-parametric Mann–Whitney test was used to compare the median values between the two groups. Statistical significance was set at *P* < 0*.*05.

## Results

### Systemic anaphylactic reactions evoked in guinea pigs by macromolecules

**Fig. 1:**
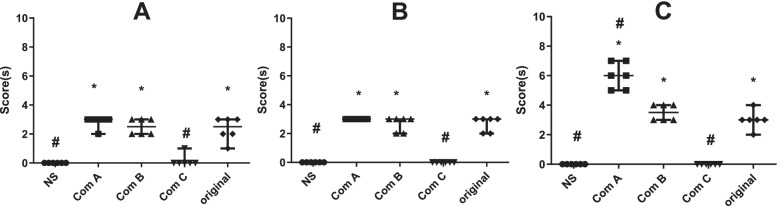
Systemic anaphylactic or anaphylactoid reactions evoked by macromolecules of TW80 (*n* = 6). **A**, Anaphylactic test in guinea pigs: Guinea pigs received 3 doses (10%, 0.5 ml) of intraperitoneal injection for sensitization and a dose (15 mg/ml, 0.5 ml) of intravenous injection for evocation; **B**, Anaphylactoid test in guinea pigs: Guinea pigs received a dose (15 mg, 1 ml) of intravenous injection for evocation without sensitization; **C**, Anaphylactoid test in rabbits: rabbits received a dose (70 mg, 0.7 ml) of intravenous injection for evocation without sensitization. The symptom with the high score appeared in 30 min after the last administration was recorded according to Table 1. A high score means a severe reaction. Com A, Component A (above 100 kDa); Com B, Component B (from 10 to 100 kDa); Com C, Component C (below 10 kDa); Ori, the original TW80; and NS, normal saline for injection. *, *P* < 0.05 vs NS; #, *P* < 0.05 vs Ori; Mann Whitney test

The original TW80 and Component A and B caused significant anaphylactic reactions, while Component C almost caused no reactions (Fig. 1A).

**Fig. 2:**
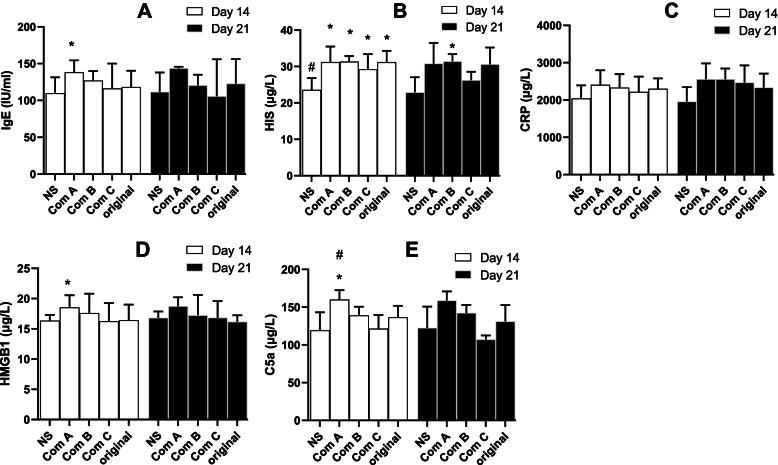
Serum factors in anaphylactic guinea pigs (mean + SD, *n* = 6). Guinea pigs received 3 doses (10%, 0.5 ml) of intraperitoneal injection for sensitization and a dose (10%, 0.5 ml) of intravenous injection for evocation. On the fourteenth day, the serum was obtained 30 min following evocation. Seven days later (Day 21), their serum samples were also collected. Serum factors, including IgE (**A**), HIS (**B**), CRP (**C**), HMGB1 (**D**), and C5a (**E**), were detected using ELISA kits. IgE, immunoglobin E; HIS, histamine; CRP, C-reactive protein; HMGB1, high mobility group protein; and C5a, complement 5a. Com A, Component A (above 100 kDa); Com B, Component B (from 10 to 100 kDa); Com C, Component C (below 10 kDa); Ori, the original TW80; and NS, normal saline for injection. *, *P* < 0.05 vs NS; #, *P* < 0.05 vs Ori; ANOVA

The levels of IgE, HIS, CRP, HMGB1 and C5a in the serum were measured (Fig. 2). Some factors were mainly elevated just after evocation (Day 14). Significant elevation of IgE (the main marker for anaphylactic reactions) was caused only by Component A after anaphylactic evocation (Fig. 2A, Day 14). However, the effector, HIS, was elevated by the original TW80 and all of its components after evocation (Fig. 2B, Day 14). CRP was not significantly affected by the original TW80 and its components neither on the day of evocation (Day 14) nor seven days after evocation (Day 21) (Fig. 2C). HMGB1 (Fig. 2D) and C5a (Fig. 2E) were elevated by Component A on the day of evocation (Day 14).

### Systemic anaphylactoid reactions evoked in guinea pigs by macromolecules

**Fig. 3:**
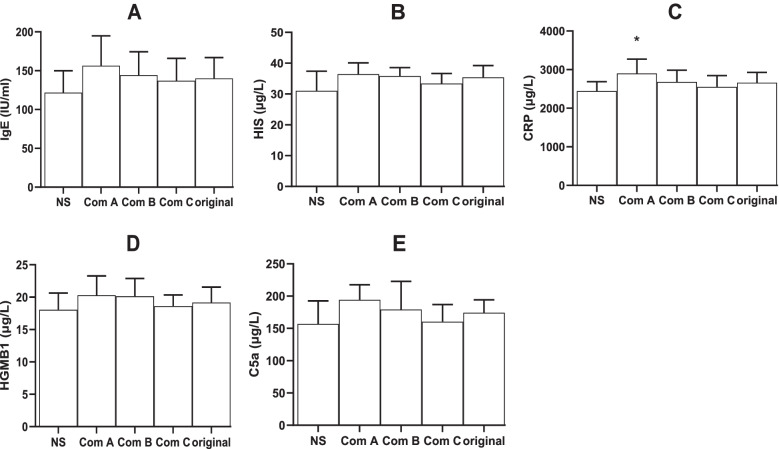
Serum factors in anaphylactoid guinea pigs (mean + SD, *n* = 6). Guinea pigs received a dose (15 mg, 1 ml) of intravenous injection for evocation without sensitization, and the serum was obtained 30 min following the evocation. Serum factors, including IgE (**A**), HIS (**B**), CRP (**C**), HMGB1 (**D**), and C5a (**E**), were detected using ELISA kits. IgE, immunoglobin E; HIS, histamine; CRP, C-reactive protein; HMGB1, high mobility group protein; and C5a, complement 5a. Com A, Component A (above 100 kDa); Com B, Component B (from 10 to 100 kDa); Com C, Component C (below 10 kDa); Ori, the original TW80; and NS, normal saline for injection. *, *P* < 0.05 vs NS; #, *P* < 0.05 vs Ori; ANOVA

The original TW80 and Component A and B caused significant anaphylactoid reactions. However, Component C almost caused no reactions (Fig. 1B). In contrast to the results shown in Fig. 2, serum factors, including IgE (Fig. 3A), HIS (Fig. 3B), HMGB1 (Fig. 3D), and C5a (Fig. 3E), were not significantly elevated by the original TW80 and its components, and only CRP (Fig. 3C) was significantly elevated by Component A.

### Anaphylactoid reaction evoked by macromolecules in rabbits

Considering that the original TW80 and its macromolecular components (A and B) caused both anaphylactic and anaphylactoid reaction in guinea pigs but with different patterns of serum factor, anaphylactoid reactions were then tested in the larger animals, rabbits. Unsurprisingly, the original TW80 and Component A and B caused significant anaphylactic reactions while Component C caused almost no reactions (Fig. 1C). Component A, which contained the macromolecules with the largest molecular weights, caused the most serious reactions.

**Fig. 4:**
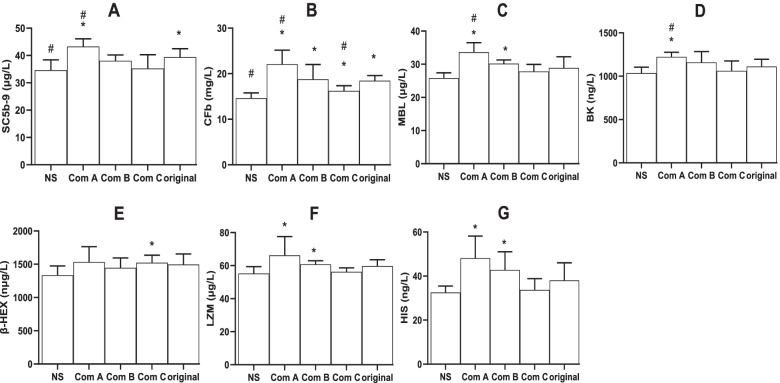
Serum factors in anaphylactoid rabbits (mean + SD, *n* = 6). Rabbits received a dose (70 mg, 0.7 ml) of intravenous injection for evocation without sensitization, and the serum was obtained 30 min following the evocation. Serum factors, including soluble complement terminal complex Sc5b-9 (Sc5b-9), complement factor B (CFb), mannose binding lectin (MBL), bradykinin (BK), β-hexosaminidase (β-HEX), lysozyme (LZM), and histamine (HIS), were detected using ELISA kits. Com A, Component A (above 100 kDa); Com B, Component B (from 10 to 100 kDa); Com C, Component C (below 10 kDa); Ori, the original TW80; and NS, normal saline for injection. *, *P* < 0.05 vs NS; #, *P* < 0.05 vs Ori; ANOVA

Next, to understand the possible mechanism underlying the systemic anaphylactoid reactions, the factors in their serum were detected. Compared to the negative control (NS), the original TW80 and Component A tended to increase the factors involved in Fig. 4, though they did not significantly affect them all. Compared to the original TW80, Component A increased the levels of SC5b-9 (Fig. 4A), CFb (Fig. 4B), BK (Fig. 4D), LZM (Fig. 4F) and HIS (Fig. 4G), while Component C did not elevate the abovementioned serum factors.

**Fig. 5:**
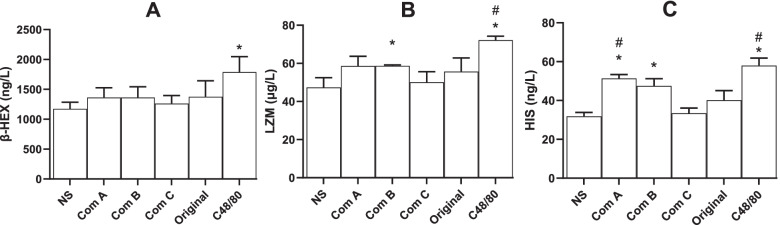
Substances released from blood leukocytes (mean + SD, *n* = 3). After guinea pig’s blood leukocytes were prepared, the leukocytes were treated with the original TW80 and its components (16 μg in 5 ml) at 37 °C for 30 min. Factors released from the leukocytes treated for 30 min were measured. β-HEX, β-hexosaminidase; LZM, lysozyme; HIS, histamine. Com A, Component A (above 100 kDa); Com B, Component B (from 10 to 100 kDa); Com C, Component C (below 10 kDa); Ori, the original TW80; and NS, normal saline for injection. C48/80 (20 μg/ml) was served as a positive control. *, *P* < 0.05 vs NS; # *P* < 0.05 vs Ori; ANOVA

To investigate whether leukocytes are involved in anaphylactic reactions, leukocytes were isolated from guinea pig blood and the released factors were measured. The results showed that Component A promoted histamine release only (Fig. C), while Component C showed no tendency to increase the factors mentioned in Fig. 5.

#### PCA reaction evoked by macromolecules in guinea pigs

**Fig. 6:**
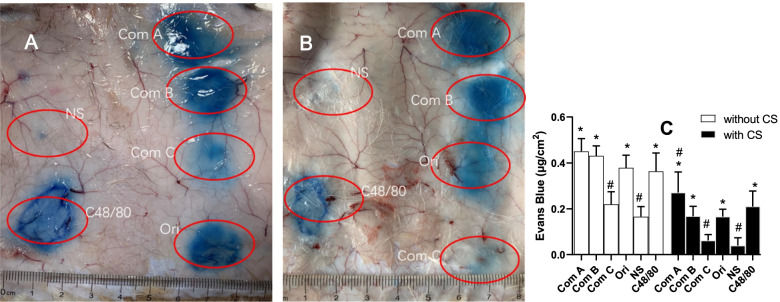
Guinea pig’s back skin was stained with Evans Blue after passive cutaneous anaphylactoid (PCA) reaction (mean + SD, *n* = 4). **A** Animal was subcutaneously administered with the original TW80 (Ori) and its components (Com A, B, and C). **B** Animal was intravenously pretreated with cromolyn sodium (CS) (50 mg/kg), and then treated with the original TW80 (Ori) and its components (10%, 0.15 ml). **C** Evans Blue in A and B (4 cm^2^) was determined. Com A, Component A (above 100 kDa); Com B, Component B (from 10 to 100 kDa); Com C, Component C (below 10 kDa); Ori, the original TW80; and NS, normal saline for injection. C48/80 (0.50 mg/ml, 0.15 ml) was served as a positive control. *, *P* < 0.05 vs NS; # *P* < 0.05 vs Ori; ANOVA

The PCA reaction is believed to be a sensitive method for detecting anaphylactoid reactions. Since anaphylactoid reactions increase vascular permeability, the leached Evans Blue from vessels can be used to assess the intensity of the reaction. According to the results in Fig. 6, normal saline for injection resulted almost no PCA reactions. However, the original TW80 and Component A and B caused strong positive PCA reactions, and the reaction caused by Component C was much weaker (Fig. 6A and C). Note that Component A caused the strongest PCA reaction among the TW80 groups. The reactions were partly prevented by cromolyn sodium (Fig. 6B and C).

**Fig. 7:**
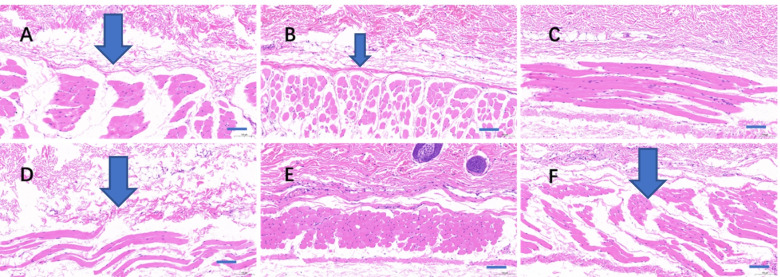
Hematoxylin–eosin (HE) staining on guinea pig’s skin section. The fields were near to the injection sites. Skin treated with Component A (**A**), Component B (**B**), Component C (**C**), the original TW80 (**D**), normal saline for injection (**E**), and C48/80 (**G**). The arrows showed hydropic degeneration. Bar = 100 μm

Considering that PCA reactions are an acute injury to the skin, regeneration rather than necrosis occurred in the area near the injection point. Local hydropic degeneration could be seen in the areas of skin with severe PCA reactions (Fig. 7), the degree of which was generally consistent with the results of PCA.

#### Pulmonary vascular permeability affected by macromolecules

**Fig. 8:**
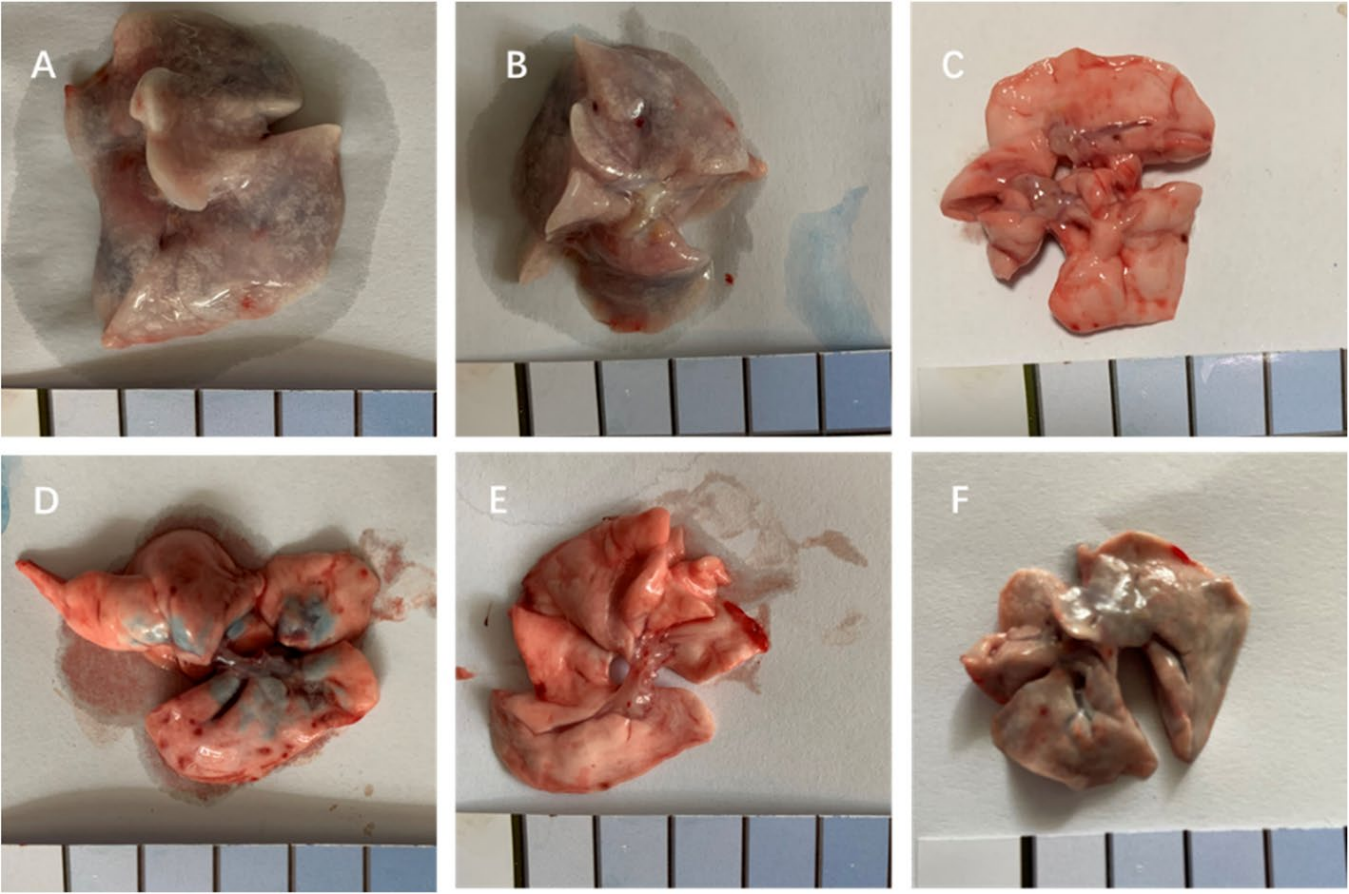
Rat lungs stained with Evans Blue after perfusing with Component A (**A**), original TW80 (**B**), Component C (**C**), cromolyn and Component A (**D**), cromolyn sodium (**E**), and normal saline for injection (**F**)

To exclude the role of blood cells mediating vascular permeability, pulmonary perfusion experiment was conducted in female rats. The results showed that the lungs treated with normal saline for injection were only slightly stained by Evans Blue (Fig. 8F), While those treated with the original TW80 and Component A were heavily stained by the dye (Fig. 8A and B). Unsurprisingly, the lungs treated with Component C (Fig. 8C) or cromolyn sodium (Fig. 8E) were almost negatively stained by the dye. The increased vascular permeability caused by Component A could be largely prevented by cromolyn sodium (Fig. 8D).

#### Vasodilation caused by macromolecules in the cat

**Fig. 9:**
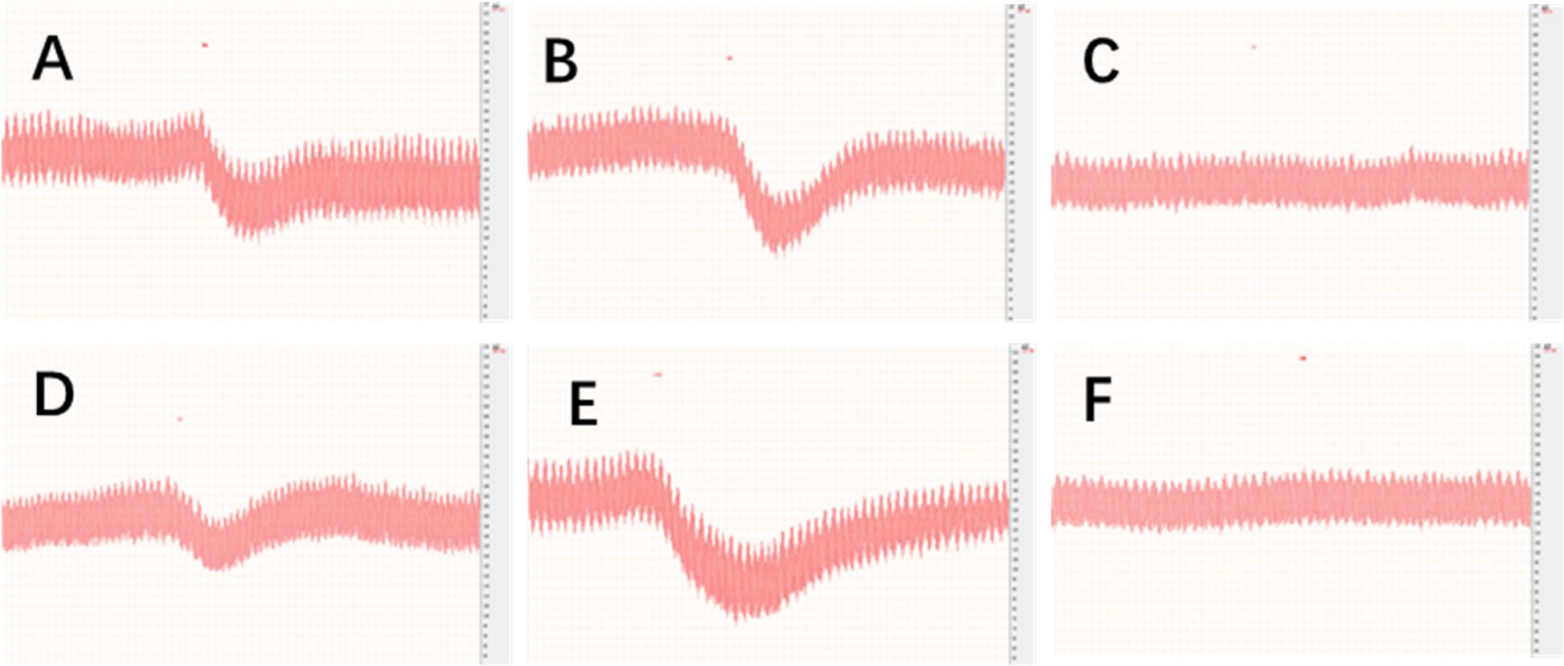
Vasodilation caused by Component A (**A**), Component B (**B**), and the original TW80 (**D**) in the cat. Histamine (**E**) was the positive control, and normal saline for injection (**F**) was the negative control. Component C (**C**) demonstrated almost no effects of vasodilation

The original TW80 and Component A and B caused strong vasodilation in the cat while Component C almost had no effects (Fig. 9).

#### Hemolysis caused by macromolecules

**Fig. 10:**
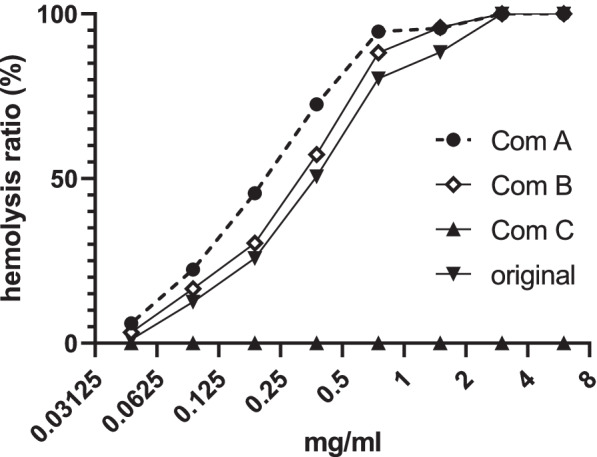
Hemolysis caused by Component A (Com A), Component B (Com B), Component C (Com C) and the original TW80 (original). The EC50 values for Com A, Com B, and original were about 0.20, 0.29, and 0.36 mg/ml, respectively. It can be deduced that the EC50 values for Com C is far bigger than 6 mg/ml

The original TW80 and Component A and B caused strong hemolysis while Component C caused no hemolysis at a concentration of ≤ 6 mg/ml (Fig. 10). Component A showed the strongest effect in hemolysis.

## Discussion

This study proves that TW80 contain macromolecular impurities. Though TW80 is a mixture of polymers, it is believed that the agent only contains small molecules with an theoretical molecular weight of 1,310 Da. However, more than 85% the substances were polymers with molecular weights > 10 kDa [15]. According to the synthesis method, TW80 must be a mixture of a series of homologues who have similar chemical structures and physicochemical properties except for different molecular weights [15]. The role of the macromolecular substances in triggering the anaphylactoid reaction has not been fully noticed before.

The original TW80 and its macromolecular components (Components A and B) caused serious anaphylactic and anaphylactoid reactions in guinea pigs. However, there was little difference between the intensity of the anaphylactoid reaction and that of the anaphylactic reaction. Considering that serum IgE both in “anaphylactic” and anaphylactoid guinea pigs was only slightly elevated by the original TW80 and its macromolecular components, the reactions in the anaphylactic experiment are more likely to be anaphylactoid reactions although the possibility of anaphylactic reaction cannot be excluded. The anaphylactoid reactions were further proved in rabbits, and Component A caused the most severe reactions. The finding that Component C partially causes anaphylactoid reactions in both guinea pigs and rabbits indicates that macromolecules are the dangerous factor triggering anaphylactoid reactions.

The anaphylactoid reactions must be associated with the release of HIS and the activation of the complement system because Component A tended to elevate the level of SC5b-9, CFB, and MBL. Moreover, the macromolecular substances of TW80 activate the bradykinin system., as shown by the elevation of BK caused by Component A in anaphylactoid reactions in rabbits. The anaphylactoid reaction is likely to be largely unassociated with the activation of mast cells because Component A barely elevated the level of β-HEX and LZM, both in vivo and in vitro, which are most probably released from mast cells [16].

Compared to anaphylactoid reactions ignited by intravenous administration, the PCA reaction is a more sensitive test with visual results [17]. The PCA reaction seldom causes animal death, which is supported by the results of the current study. The advantage of the PCA reaction is that the degree of the anaphylactoid reaction can be quantitatively evaluated by the exudation of Evans Blue, and the principle of the test is to evaluate vascular permeability. The results of PCA supported that macromolecular substances are the components responsible for anaphylactoid reactions, and the reaction could be partly prevented by cromolyn sodium. Micropathological observations illustrated that the PCA reaction caused by macromolecular substances is associated with hydropic degeneration.

The macromolecular substances from TW80 enhanced the vascular permeability in the pulmonary perfusion experiments. Considering that blood cells, including leukocytes, were removed by perfusate, and given that large molecules are unlikely to cross the vascular wall into the interstitial space to activate mast cells where they reside, it can be deduced that the increase in vascular permeability is likely to have resulted from a direct effect on epithelia.

Though cromolyn sodium is a mast cell stabilizer with anti-inflammatory activity, it also has other pharmacological effects, including reducing vessel permeability [18]. Thus, it is understandable that cromolyn sodium partially prevents the PCA reactions and the increase in vascular permeability caused by macromolecular substances from TW80. Furthermore, the macromolecular substances can dilate vessels to facilitate hydropic degeneration and hypotension, which are usually part of anaphylactoid symptoms [19, 20].

Additionally, the original TW80 and its macromolecular components caused hemolysis at low concentrations, while Component C did not, even at 6 mg/ml, an impossible concentration in serum by intravenous administration. Thus, the macromolecular substances also demonstrate hemolytic toxicity.

## Conclusion

Taken together, these results suggest that macromolecular substances > 10 kDa are one of the main risk factors responsible for anaphylactoid reactions caused by TW80, which can be removed by ultrafiltration. The direct increase in vascular permeability participates in the anaphylactoid reactions ignited by TW80, although a role for mast cell degranulation cannot be excluded as one of the main mediators of the reaction.

## Supplementary Information

**Additional file 1.**

## Data Availability

All data generated or analysed during this study are included in this published article [and its supplementary information files].
