# Supplementary material for: Macromolecules in polysorbate 80 for injection: an important cause of anaphylactoid reactions
Source: BMC Pharmacol Toxicol. 2022 Jul 18;23:52. doi: 10.1186/s40360-022-00591-5 (PMC9295270; doi:10.1186/s40360-022-00591-5)
Supplement: Supplementary file 1 — Additional file 1. [file 40360_2022_591_MOESM1_ESM.zip › supplement of raw data.docx]

目录

[1 Raw data for Figure 1 2](#_Toc100955854)

[1.1 Raw data for Fig 1A 2](#_Toc100955855)

[1.2 Raw data for Fig 1B 2](#_Toc100955856)

[1.3 Raw data for Fig 1C 2](#_Toc100955857)

[2 Raw data for Figure 2 3](#_Toc100955858)

[2.1 Raw data for Fig 2A 3](#_Toc100955859)

[2.2 Raw data for Fig 2B 3](#_Toc100955860)

[2.3 Raw data for Fig 2C 4](#_Toc100955861)

[2.4 Raw data for Fig 2D 4](#_Toc100955862)

[2.5 Raw data for Fig 2E 4](#_Toc100955863)

[3 Raw data for Figure 3 5](#_Toc100955864)

[3.1 Raw data for Fig 3A 5](#_Toc100955865)

[3.2 Raw data for Fig 3B 5](#_Toc100955866)

[3.3 Raw data for Fig 3C 5](#_Toc100955867)

[3.4 Raw data for Fig 3D 6](#_Toc100955868)

[3.5 Raw data for Fig 3E 6](#_Toc100955869)

[4 Raw data for Figure 4 6](#_Toc100955870)

[4.1 Raw data for Fig 4A 6](#_Toc100955871)

[4.2 Raw data for Fig 4B 6](#_Toc100955872)

[4.3 Raw data for Fig 4C 7](#_Toc100955873)

[4.4 Raw data for Fig 4D 7](#_Toc100955874)

[4.5 Raw data for Fig 4E 7](#_Toc100955875)

[4.6 Raw data for Fig 4F 7](#_Toc100955876)

[4.7 Raw data for Fig 4G 8](#_Toc100955877)

[5 Raw data for Figure 5 8](#_Toc100955878)

[5.1 Raw data for Fig 5A 8](#_Toc100955879)

[5.2 Raw data for Fig 5B 8](#_Toc100955880)

[5.3 Raw data for Fig 5C 8](#_Toc100955881)

[6 Raw data for Figure 6 9](#_Toc100955882)

[6.1 Raw data for 6A 9](#_Toc100955883)

[6.2 Raw data for 6B 13](#_Toc100955884)

[6.3 Raw data for Fig 6C without cromolyn sodium (CS): from 6.1 16](#_Toc100955885)

[6.4 Raw data for Fig 6C with cromolyn sodium (CS) (50 mg/kg) : from 6.2 16](#_Toc100955886)

[7 Raw data for Figure 10 17](#_Toc100955887)

# 1 Raw data for Figure 1

## 1.1 Raw data for Fig 1A

| anaphyactic in guinea pig | 1 | 2 | 3 | 4 | 5 | 6 | P1 vs NS | P2 Vs Original |
| --- | --- | --- | --- | --- | --- | --- | --- | --- |
| NS | 0 | 0 | 0 | 0 | 0 | 0 | 1 | 0.02 |
| Com A | 3 | 3 | 3 | 3 | 2 | 3 | 0.01 | 0.211 |
| Com B | 2 | 3 | 3 | 2 | 2 | 3 | 0.02 | 0.789 |
| Com C | 0 | 1 | 0 | 0 | 0 | 0 | 0.317 | 0.03 |
| original | 3 | 2 | 1 | 3 | 2 | 3 | 0.02 | 1 |

## 1.2 Raw data for Fig 1B

| anaphyactic in guinea pig | 1 | 2 | 3 | 4 | 5 | 6 | P1 vs NS | P2 Vs Original |
| --- | --- | --- | --- | --- | --- | --- | --- | --- |
| NS | 0 | 0 | 0 | 0 | 0 | 0 | 1 | 0.02 |
| Com A | 3 | 3 | 3 | 3 | 3 | 3 | 0.01 | 0.138 |
| Com B | 3 | 3 | 2 | 2 | 3 | 3 | 0.02 | 1 |
| Com C | 0 | 0 | 0 | 0 | 0 | 0 | 1 | 0.02 |
| original | 3 | 2 | 3 | 2 | 3 | 3 | 0.02 | 1 |

## 1.3 Raw data for Fig 1C

| anaphyactic in guinea pig | 1 | 2 | 3 | 4 | 5 | 6 | P1 vs NS | P2 Vs Original |
| --- | --- | --- | --- | --- | --- | --- | --- | --- |
| NS | 0 | 0 | 0 | 0 | 0 | 0 | 1 | 0.02 |
| Com A | 5 | 7 | 6 | 5 | 6 | 7 | 0.02 | 0.03 |
| Com B | 3 | 4 | 3 | 3 | 4 | 4 | 0.02 | 0.171 |
| Com C | 0 | 0 | 0 | 0 | 0 | 0 | 1 | 0.02 |
| original | 3 | 2 | 3 | 3 | 4 | 3 | 0.02 | 1 |

# 2 Raw data for Figure 2

## 2.1 Raw data for Fig 2A

| IgE | 14Day |  |  |  |  |  |  |  |  |  |
| --- | --- | --- | --- | --- | --- | --- | --- | --- | --- | --- |
| IU/ml | 1 | 2 | 3 | 4 | 5 | 6 | mean | SD | P1 vs NS | P2 vs Ori |
| NS | 109.37 | 86.22 | 128.81 | 82.27 | 119.06 | 133.41 | 109.86 | 21.53 | 1.00 | 0.52 |
| Com A | 159.31 | 114.99 | 123.78 | 141.61 | 144.60 | 145.25 | 138.26 | 16.09 | 0.03 | 0.10 |
| Com B | 140.77 | 122.65 | 136.88 | 104.64 | 132.51 | 124.62 | 127.01 | 12.98 | 0.13 | 0.42 |
| Com C | 121.63 | 124.80 | 133.29 | 138.02 | 48.00 | 131.44 | 116.20 | 33.93 | 0.71 | 0.90 |
| original | 109.79 | 123.30 | 107.45 | 121.03 | 156.92 | 90.89 | 118.23 | 22.21 | 0.52 | 1.00 |

| 21Day |  |  |  |  |  |  |  |
| --- | --- | --- | --- | --- | --- | --- | --- |
|  | 1 | 2 | 3 | mean | SD | P1 vs NS | P2 vs Ori |
| NS | 82.27 | 119.06 | 133.41 | 111.58 | 26.38 | 1.00 | 0.67 |
| Com A | 141.61 | 144.60 | 145.25 | 143.82 | 1.94 | 0.10 | 0.34 |
| Com B | 104.64 | 132.51 | 124.62 | 120.59 | 14.37 | 0.63 | 0.92 |
| Com C | 138.02 | 48.00 | 131.44 | 105.82 | 50.18 | 0.87 | 0.65 |
| original | 121.03 | 156.92 | 90.89 | 122.95 | 33.06 | 0.67 | 1.00 |

## 2.2 Raw data for Fig 2B

| HIS | 14Day |  |  |  |  |  |  |  |  |  |
| --- | --- | --- | --- | --- | --- | --- | --- | --- | --- | --- |
| μg/L | 1 | 2 | 3 | 4 | 5 | 6 | mean | SD | P1 vs NS | P2 vs Ori |
| NS | 22.16 | 23.17 | 27.49 | 21.91 | 27.45 | 19.35 | 23.59 | 3.26 | 1.000 | 0.002 |
| Com A | 35.67 | 30.33 | 28.91 | 30.96 | 25.10 | 36.41 | 31.23 | 4.25 | 0.006 | 0.988 |
| Com B | 32.83 | 30.90 | 30.19 | 29.21 | 33.13 | 31.89 | 31.36 | 1.53 | 0.000 | 0.909 |
| Com C | 36.16 | 30.33 | 30.13 | 24.64 | 25.26 | 28.89 | 29.24 | 4.17 | 0.026 | 0.376 |
| original | 31.50 | 33.13 | 30.67 | 31.87 | 25.54 | 34.46 | 31.19 | 3.07 | 0.002 | 1.000 |

| 21Day |  |  |  |  |  |  |  |
| --- | --- | --- | --- | --- | --- | --- | --- |
|  | 1 | 2 | 3 | mean | SD | P1 vs NS | P2 vs Ori |
| NS | 21.91 | 27.45 | 19.35 | 22.91 | 4.14 | 1.000 | 0.097 |
| Com A | 30.96 | 25.10 | 36.41 | 30.82 | 5.66 | 0.122 | 0.965 |
| Com B | 29.21 | 33.13 | 31.89 | 31.41 | 2.00 | 0.033 | 0.799 |
| Com C | 24.64 | 25.26 | 28.89 | 26.26 | 2.30 | 0.286 | 0.215 |
| original | 31.87 | 25.54 | 34.46 | 30.62 | 4.59 | 0.097 | 1.000 |

## 2.3 Raw data for Fig 2C

| CRP | 14Day |  |  |  |  |  |  |  |  |  |
| --- | --- | --- | --- | --- | --- | --- | --- | --- | --- | --- |
| μg/L | 1 | 2 | 3 | 4 | 5 | 6 | mean | SD | P1 vs NS | P2 vs Ori |
| NS | 2506.90 | 2096.81 | 1763.67 | 2124.29 | 2234.24 | 1516.30 | 2040.37 | 351.19 | 1.000 | 0.181 |
| Com A | 2551.98 | 1859.33 | 2365.07 | 2986.26 | 2551.98 | 2138.59 | 2408.87 | 387.64 | 0.115 | 0.595 |
| Com B | 2314.50 | 2233.14 | 1754.88 | 2389.26 | 2401.35 | 2888.41 | 2330.26 | 363.54 | 0.190 | 0.884 |
| Com C | 1792.26 | 2036.34 | 2073.72 | 2968.67 | 2369.47 | 2067.12 | 2217.93 | 410.92 | 0.440 | 0.684 |
| original | 2422.24 | 2026.44 | 2362.87 | 2159.48 | 2079.22 | 2764.17 | 2302.40 | 274.83 | 0.181 | 1.000 |

| 21Day |  |  |  |  |  |  |  |
| --- | --- | --- | --- | --- | --- | --- | --- |
|  | 1 | 2 | 3 | mean | SD | P1 vs NS | P2 vs Ori |
| NS | 2124.29 | 2234.24 | 1516.30 | 1958.28 | 386.69 | 1.000 | 0.293 |
| Com A | 2986.26 | 2551.98 | 2138.59 | 2558.94 | 423.88 | 0.144 | 0.529 |
| Com B | 2389.26 | 2401.35 | 2888.41 | 2559.68 | 284.76 | 0.096 | 0.453 |
| Com C | 2968.67 | 2369.47 | 2067.12 | 2468.42 | 458.85 | 0.215 | 0.715 |
| original | 2159.48 | 2079.22 | 2764.17 | 2334.29 | 374.45 | 0.293 | 1.000 |

## 2.4 Raw data for Fig 2D

| HGMB1 | 14Day |  |  |  |  |  |  |  |  |  |
| --- | --- | --- | --- | --- | --- | --- | --- | --- | --- | --- |
| μg/L | 1 | 2 | 3 | 4 | 5 | 6 | mean | SD | P1 vs NS | P2 vs Ori |
| NS | 16.22 | 15.06 | 16.40 | 16.53 | 15.98 | 17.98 | 16.36 | 0.95 | 1.000 | 0.946 |
| Com A | 20.97 | 15.44 | 18.70 | 19.57 | 19.63 | 17.02 | 18.56 | 2.00 | 0.036 | 0.142 |
| Com B | 20.70 | 13.61 | 19.46 | 18.07 | 13.60 | 20.13 | 17.60 | 3.21 | 0.388 | 0.506 |
| Com C | 11.51 | 16.71 | 18.82 | 13.98 | 19.48 | 17.04 | 16.26 | 3.01 | 0.937 | 0.912 |
| original | 20.64 | 16.58 | 12.87 | 17.19 | 16.26 | 15.09 | 16.44 | 2.56 | 0.946 | 1.000 |

| 21Day |  |  |  |  |  |  |  |
| --- | --- | --- | --- | --- | --- | --- | --- |
|  | 1 | 2 | 3 | mean | SD | P1 vs NS | P2 vs Ori |
| NS | 16.53 | 15.98 | 17.98 | 16.83 | 1.03 | 1.000 | 0.488 |
| Com A | 19.57 | 19.63 | 17.02 | 18.74 | 1.49 | 0.141 | 0.072 |
| Com B | 18.07 | 13.60 | 20.13 | 17.27 | 3.34 | 0.839 | 0.619 |
| Com C | 13.98 | 19.48 | 17.04 | 16.83 | 2.75 | 0.998 | 0.721 |
| original | 17.19 | 16.26 | 15.09 | 16.18 | 1.05 | 0.488 | 1.000 |

## 2.5 Raw data for Fig 2E

| C5a | 14Day |  |  |  |  |  |  |  |  |  |
| --- | --- | --- | --- | --- | --- | --- | --- | --- | --- | --- |
| ng/ml | 1 | 2 | 3 | 4 | 5 | 6 | mean | SD | P1 vs NS | P2 vs Ori |
| NS | 87.64 | 129.63 | 131.08 | 124.74 | 149.65 | 93.60 | 119.39 | 23.91 | 1.000 | 0.169 |
| Com A | 173.01 | 143.59 | 167.37 | 171.40 | 157.98 | 147.56 | 160.15 | 12.50 | 0.004 | 0.015 |
| Com B | 146.22 | 120.12 | 140.74 | 132.10 | 142.51 | 152.88 | 139.09 | 11.52 | 0.099 | 0.751 |
| Com C | 143.64 | 120.66 | 143.10 | 101.92 | 112.55 | 107.18 | 121.51 | 18.03 | 0.866 | 0.149 |
| original | 144.66 | 136.34 | 144.71 | 117.22 | 120.28 | 156.10 | 136.55 | 15.18 | 0.169 | 1.000 |

| 21Day |  |  |  |  |  |  |  |
| --- | --- | --- | --- | --- | --- | --- | --- |
|  | 1 | 2 | 3 | mean | SD | P1 vs NS | P2 vs Ori |
| NS | 124.74 | 149.65 | 93.60 | 122.66 | 28.09 | 1.000 | 0.698 |
| Com A | 171.40 | 157.98 | 147.56 | 158.98 | 11.95 | 0.108 | 0.123 |
| Com B | 132.10 | 142.51 | 152.88 | 142.49 | 10.39 | 0.315 | 0.460 |
| Com C | 101.92 | 112.55 | 107.18 | 107.22 | 5.32 | 0.402 | 0.135 |
| original | 117.22 | 120.28 | 156.10 | 131.20 | 21.62 | 0.698 | 1.000 |

# 3 Raw data for Figure 3

## 3.1 Raw data for Fig 3A

| IgE | guinea pig | |  |  |  |  |  |  |  |  |
| --- | --- | --- | --- | --- | --- | --- | --- | --- | --- | --- |
| IU/ml | 1 | 2 | 3 | 4 | 5 | 6 | mean | SD | P1 vs NS | P2 vs Ori |
| NS | 109.19 | 99.74 | 124.50 | 126.77 | 96.51 | 173.48 | 121.70 | 28.24 | 1.000 | 0.277 |
| Com A | 98.12 | 148.66 | 135.68 | 159.67 | 198.90 | 197.83 | 156.48 | 38.52 | 0.105 | 0.410 |
| Com B | 116.07 | 102.01 | 145.37 | 151.77 | 174.92 | 175.22 | 144.23 | 30.12 | 0.211 | 0.803 |
| Com C | 109.43 | 93.82 | 152.07 | 142.50 | 163.68 | 161.04 | 137.09 | 28.89 | 0.373 | 0.860 |
| original | 103.57 | 109.19 | 151.41 | 148.66 | 166.43 | 160.75 | 140.00 | 26.87 | 0.277 | 1.000 |

## 3.2 Raw data for Fig 3B

| HIS | guinea pig | |  |  |  |  |  |  |  |  |
| --- | --- | --- | --- | --- | --- | --- | --- | --- | --- | --- |
| μg/L | 1 | 2 | 3 | 4 | 5 | 6 | mean | SD | P1 vs NS | P2 vs Ori |
| NS | 27.36 | 21.69 | 35.34 | 40.17 | 30.97 | 30.64 | 31.03 | 6.37 | 1.000 | 0.181 |
| Com A | 35.56 | 30.82 | 35.23 | 36.96 | 42.08 | 37.92 | 36.43 | 3.69 | 0.103 | 0.644 |
| Com B | 33.39 | 34.65 | 32.31 | 36.81 | 38.89 | 38.75 | 35.80 | 2.78 | 0.124 | 0.837 |
| Com C | 32.64 | 32.19 | 32.56 | 29.27 | 34.95 | 38.86 | 33.41 | 3.23 | 0.433 | 0.355 |
| original | 29.14 | 34.40 | 34.28 | 39.53 | 39.18 | 35.83 | 35.39 | 3.82 | 0.181 | 1.000 |

## 3.3 Raw data for Fig 3C

| CRP | guinea pig | |  |  |  |  |  |  |  |  |
| --- | --- | --- | --- | --- | --- | --- | --- | --- | --- | --- |
| μg/L | 1 | 2 | 3 | 4 | 5 | 6 | mean | SD | P1 vs NS | P2 vs Ori |
| NS | 2352.98 | 2277.12 | 2138.59 | 2455.23 | 2669.62 | 2778.47 | 2445.33 | 241.79 | 1.000 | 0.175 |
| Com A | 2840.03 | 2580.56 | 2371.67 | 3075.32 | 3285.31 | 3255.63 | 2901.42 | 371.60 | 0.030 | 0.226 |
| Com B | 2917.00 | 2495.91 | 2224.34 | 2648.73 | 3074.22 | 2734.49 | 2682.45 | 302.32 | 0.164 | 0.895 |
| Com C | 2606.95 | 2399.16 | 2260.62 | 2801.55 | 2971.97 | 2266.12 | 2551.06 | 293.82 | 0.512 | 0.517 |
| original | 2317.80 | 2591.56 | 2621.24 | 2544.28 | 2767.47 | 3118.20 | 2660.09 | 267.65 | 0.175 | 1.000 |

## 3.4 Raw data for Fig 3D

| HGMB1 | guinea pig | |  |  |  |  |  |  |  |  |
| --- | --- | --- | --- | --- | --- | --- | --- | --- | --- | --- |
| μg/L | 1 | 2 | 3 | 4 | 5 | 6 | mean | SD | P1 vs NS | P2 vs Ori |
| NS | 17.75 | 16.76 | 17.84 | 14.18 | 20.72 | 21.11 | 18.06 | 2.58 | 1.000 | 0.455 |
| Com A | 18.71 | 18.99 | 18.88 | 21.74 | 17.75 | 25.73 | 20.30 | 2.98 | 0.193 | 0.485 |
| Com B | 21.61 | 17.22 | 16.42 | 23.58 | 20.92 | 21.08 | 20.14 | 2.75 | 0.207 | 0.531 |
| Com C | 18.28 | 19.67 | 15.96 | 21.05 | 18.91 | 17.88 | 18.62 | 1.72 | 0.664 | 0.658 |
| original | 17.75 | 16.76 | 17.84 | 14.18 | 20.72 | 21.11 | 18.06 | 2.58 | 1.000 | 0.455 |

## 3.5 Raw data for Fig 3E

| C5a | guinea pig | |  |  |  |  |  |  |  |  |
| --- | --- | --- | --- | --- | --- | --- | --- | --- | --- | --- |
| μg/L | 1 | 2 | 3 | 4 | 5 | 6 | mean | SD | P1 vs NS | P2 vs Ori |
| NS | 128.07 | 105.68 | 178.76 | 151.59 | 175.48 | 202.38 | 156.99 | 35.71 | 1.000 | 0.323 |
| Com A | 198.41 | 169.09 | 163.51 | 203.51 | 222.89 | 208.61 | 194.34 | 23.27 | 0.057 | 0.141 |
| Com B | 194.22 | 145.20 | 165.28 | 204.74 | 122.59 | 243.03 | 179.18 | 43.60 | 0.358 | 0.811 |
| Com C | 150.57 | 114.91 | 158.73 | 192.99 | 169.79 | 175.43 | 160.40 | 26.62 | 0.855 | 0.328 |
| original | 175.48 | 179.13 | 136.28 | 178.49 | 195.40 | 181.39 | 174.36 | 19.91 | 0.323 | 1.000 |

# 4 Raw data for Figure 4

## 4.1 Raw data for Fig 4A

| SC5b-9 | μg/L | |  |  |  |  |  |  |  |  |
| --- | --- | --- | --- | --- | --- | --- | --- | --- | --- | --- |
|  | 1 | 2 | 3 | 4 | 5 | 6 | mean | SD | P1 vs NS | P2 vs Ori |
| NS | 31.32 | 32.55 | 32.68 | 33.09 | 41.40 | 36.63 | 34.61 | 3.78 | 1.000 | 0.036 |
| Com A | 44.13 | 43.04 | 40.18 | 44.81 | 47.40 | 40.04 | 43.27 | 2.84 | 0.001 | 0.047 |
| Com B | 40.59 | 38.41 | 36.36 | 35.14 | 40.31 | 37.45 | 38.04 | 2.16 | 0.082 | 0.393 |
| Com C | 40.99 | 27.23 | 36.23 | 31.59 | 39.09 | 36.23 | 35.23 | 5.04 | 0.816 | 0.113 |
| Ori | 40.18 | 37.18 | 37.45 | 40.45 | 44.67 | 36.50 | 39.41 | 3.05 | 0.036 | 1.000 |

## 4.2 Raw data for Fig 4B

| CFb | mg/L | |  |  |  |  |  |  |  |  |
| --- | --- | --- | --- | --- | --- | --- | --- | --- | --- | --- |
|  | 1 | 2 | 3 | 4 | 5 | 6 | mean | SD | P1 vs NS | P2 vs Ori |
| NS | 16.79 | 14.70 | 13.71 | 13.59 | 14.82 | 14.13 | 14.62 | 1.17 | 1.000 | 0.000 |
| Com A | 21.38 | 20.21 | 17.52 | 26.31 | 24.07 | 23.02 | 22.08 | 3.09 | 0.000 | 0.022 |
| Com B | 19.30 | 16.73 | 13.40 | 21.51 | 22.08 | 19.63 | 18.77 | 3.24 | 0.014 | 0.828 |
| Com C | 16.34 | 14.10 | 15.79 | 16.91 | 17.06 | 17.06 | 16.21 | 1.15 | 0.039 | 0.007 |
| Ori | 17.91 | 18.12 | 19.00 | 19.60 | 19.51 | 16.64 | 18.46 | 1.13 | 0.000 | 1.000 |

## 4.3 Raw data for Fig 4C

| MBL | μg/L | |  |  |  |  |  |  |  |  |
| --- | --- | --- | --- | --- | --- | --- | --- | --- | --- | --- |
|  | 1 | 2 | 3 | 4 | 5 | 6 | mean | SD | P1 vs NS | P2 vs Ori |
| NS | 27.60 | 24.09 | 27.32 | 23.88 | 25.42 | 26.62 | 25.82 | 1.61 | 1.000 | 0.074 |
| Com A | 32.58 | 31.88 | 30.90 | 31.88 | 38.20 | 36.24 | 33.61 | 2.91 | 0.000 | 0.027 |
| Com B | 31.32 | 28.09 | 30.20 | 30.48 | 30.76 | 30.20 | 30.17 | 1.10 | 0.000 | 0.393 |
| Com C | 27.95 | 27.67 | 27.32 | 27.39 | 31.60 | 25.07 | 27.83 | 2.11 | 0.093 | 0.537 |
| Ori | 29.85 | 27.60 | 24.44 | 30.41 | 34.20 | 26.76 | 28.87 | 3.39 | 0.074 | 1.000 |

## 4.4 Raw data for Fig 4D

| BK | ng/L | |  |  |  |  |  |  |  |  |
| --- | --- | --- | --- | --- | --- | --- | --- | --- | --- | --- |
|  | 1 | 2 | 3 | 4 | 5 | 6 | mean | SD | P1 vs NS | P2 vs Ori |
| NS | 1040.00 | 944.69 | 1011.88 | 1140.00 | 999.38 | 1080.63 | 1036.09 | 67.95 | 1.000 | 0.120 |
| Com A | 1207.19 | 1236.88 | 1227.50 | 1203.02 | 1153.02 | 1314.48 | 1223.68 | 53.15 | 0.000 | 0.020 |
| Com B | 1161.88 | 971.25 | 1357.19 | 1197.81 | 1105.63 | 1160.31 | 1159.01 | 125.61 | 0.061 | 0.459 |
| Com C | 1138.44 | 972.81 | 1122.81 | 1179.06 | 880.63 | 1077.50 | 1061.88 | 113.43 | 0.643 | 0.412 |
| Ori | 1027.50 | 1002.50 | 1222.81 | 1172.81 | 1105.63 | 1136.88 | 1111.35 | 84.58 | 0.120 | 1.000 |

## 4.5 Raw data for Fig 4E

| beta-Hex | ng/L | |  |  |  |  |  |  |  |  |
| --- | --- | --- | --- | --- | --- | --- | --- | --- | --- | --- |
|  | 1 | 2 | 3 | 4 | 5 | 6 | mean | SD | P1 vs NS | P2 vs Ori |
| NS | 1547.92 | 1122.92 | 1302.08 | 1297.92 | 1406.25 | 1331.25 | 1334.72 | 139.90 | 1.000 | 0.089 |
| Com A | 1122.92 | 1560.42 | 1664.58 | 1460.42 | 1806.25 | 1577.08 | 1531.94 | 231.56 | 0.104 | 0.763 |
| Com B | 1256.25 | 1447.92 | 1431.25 | 1639.58 | 1585.42 | 1310.42 | 1445.14 | 149.34 | 0.216 | 0.575 |
| Com C | 1456.25 | 1568.75 | 1589.58 | 1335.42 | 1656.25 | 1531.25 | 1522.92 | 113.13 | 0.028 | 0.746 |
| Ori | 1643.75 | 1356.25 | 1364.58 | 1710.42 | 1364.58 | 1539.58 | 1496.53 | 157.34 | 0.089 | 1.000 |

## 4.6 Raw data for Fig 4F

| LZM | μg/L | |  |  |  |  |  |  |  |  |
| --- | --- | --- | --- | --- | --- | --- | --- | --- | --- | --- |
|  | 1 | 2 | 3 | 4 | 5 | 6 | mean | SD | P1 vs NS | P2 vs Ori |
| NS | 57.89 | 59.87 | 55.48 | 52.85 | 57.02 | 48.25 | 55.23 | 4.15 | 1.000 | 0.084 |
| Com A | 57.02 | 88.38 | 60.09 | 66.67 | 64.04 | 61.18 | 66.23 | 11.35 | 0.050 | 0.211 |
| Com B | 60.96 | 61.84 | 59.21 | 59.65 | 64.47 | 58.33 | 60.75 | 2.21 | 0.017 | 0.575 |
| Com C | 57.68 | 56.36 | 56.80 | 56.36 | 58.55 | 51.54 | 56.21 | 2.44 | 0.627 | 0.094 |
| Ori | 65.13 | 61.18 | 57.24 | 58.33 | 54.17 | 62.06 | 59.69 | 3.89 | 0.084 | 1.000 |

## 4.7 Raw data for Fig 4G

| HIS | ng/L | |  |  |  |  |  |  |  |  |
| --- | --- | --- | --- | --- | --- | --- | --- | --- | --- | --- |
|  | 1 | 2 | 3 | 4 | 5 | 6 | mean | SD | P1 vs NS | P2 vs Ori |
| NS | 32.65 | 28.75 | 34.48 | 35.63 | 34.48 | 29.09 | 32.52 | 2.94 | 1.000 | 0.145 |
| Com A | 48.13 | 40.91 | 32.65 | 60.63 | 52.49 | 54.09 | 48.15 | 10.03 | 0.004 | 0.082 |
| Com B | 42.28 | 38.15 | 30.70 | 50.42 | 53.41 | 41.71 | 42.78 | 8.25 | 0.017 | 0.334 |
| Com C | 38.50 | 24.16 | 33.45 | 35.97 | 37.01 | 33.22 | 33.72 | 5.11 | 0.628 | 0.293 |
| Ori | 36.09 | 26.11 | 32.42 | 47.67 | 44.00 | 41.82 | 38.02 | 8.00 | 0.145 | 1.000 |

# 5 Raw data for Figure 5

## 5.1 Raw data for Fig 5A

| beta-Hex | ng/L | |  |  |  |  |  |
| --- | --- | --- | --- | --- | --- | --- | --- |
|  | 1 | 2 | 3 | mean | SD | P1 vs NS | P2 vs Ori |
| NS | 1302.08 | 1118.75 | 1102.08 | 1174.31 | 110.97 | 1.000 | 0.293 |
| Com A | 1510.42 | 1389.58 | 1189.58 | 1363.19 | 162.04 | 0.171 | 0.948 |
| Com B | 1247.92 | 1277.08 | 1568.75 | 1364.58 | 177.41 | 0.190 | 0.955 |
| Com C | 1418.75 | 1172.92 | 1189.58 | 1260.42 | 137.37 | 0.446 | 0.542 |
| Ori | 1193.75 | 1252.08 | 1681.25 | 1375.69 | 266.22 | 0.293 | 1.000 |
| C48/80 | 1568.75 | 2072.92 | 1722.92 | 1788.19 | 258.34 | 0.019 | 0.126 |

## 5.2 Raw data for Fig 5B

| LZM | μg/L | |  |  |  |  |  |
| --- | --- | --- | --- | --- | --- | --- | --- |
|  | 1 | 2 | 3 | mean | SD | P1 vs NS | P2 vs Ori |
| NS | 44.96 | 53.29 | 43.86 | 47.37 | 5.16 | 1.000 | 0.177 |
| Com A | 55.92 | 64.47 | 55.48 | 58.63 | 5.07 | 0.054 | 0.594 |
| Com B | 58.55 | 58.33 | 59.21 | 58.70 | 0.46 | 0.019 | 0.509 |
| Com C | 51.10 | 44.08 | 55.04 | 50.07 | 5.55 | 0.570 | 0.342 |
| Ori | 48.25 | 62.50 | 56.36 | 55.70 | 7.15 | 0.177 | 1.000 |
| C48/80 | 74.12 | 72.37 | 69.96 | 72.15 | 2.09 | 0.002 | 0.019 |

## 5.3 Raw data for Fig 5C

| HIS | ng/L | |  |  |  |  |  |
| --- | --- | --- | --- | --- | --- | --- | --- |
|  | 1 | 2 | 3 | mean | SD | P1 vs NS | P2 vs Ori |
| NS | 29.78 | 33.80 | 31.96 | 31.85 | 2.01 | 1.000 | 0.054 |
| Com A | 51.00 | 53.52 | 49.51 | 51.34 | 2.03 | 0.000 | 0.022 |
| Com B | 46.30 | 44.46 | 51.69 | 47.48 | 3.76 | 0.003 | 0.110 |
| Com C | 34.71 | 35.17 | 30.36 | 33.41 | 2.66 | 0.461 | 0.106 |
| Ori | 43.54 | 34.48 | 42.40 | 40.14 | 4.93 | 0.054 | 1.000 |
| C48/80 | 53.75 | 61.32 | 58.91 | 57.99 | 3.87 | 0.000 | 0.008 |

# 6 Raw data for Figure 6

## 6.1 Raw data for 6A


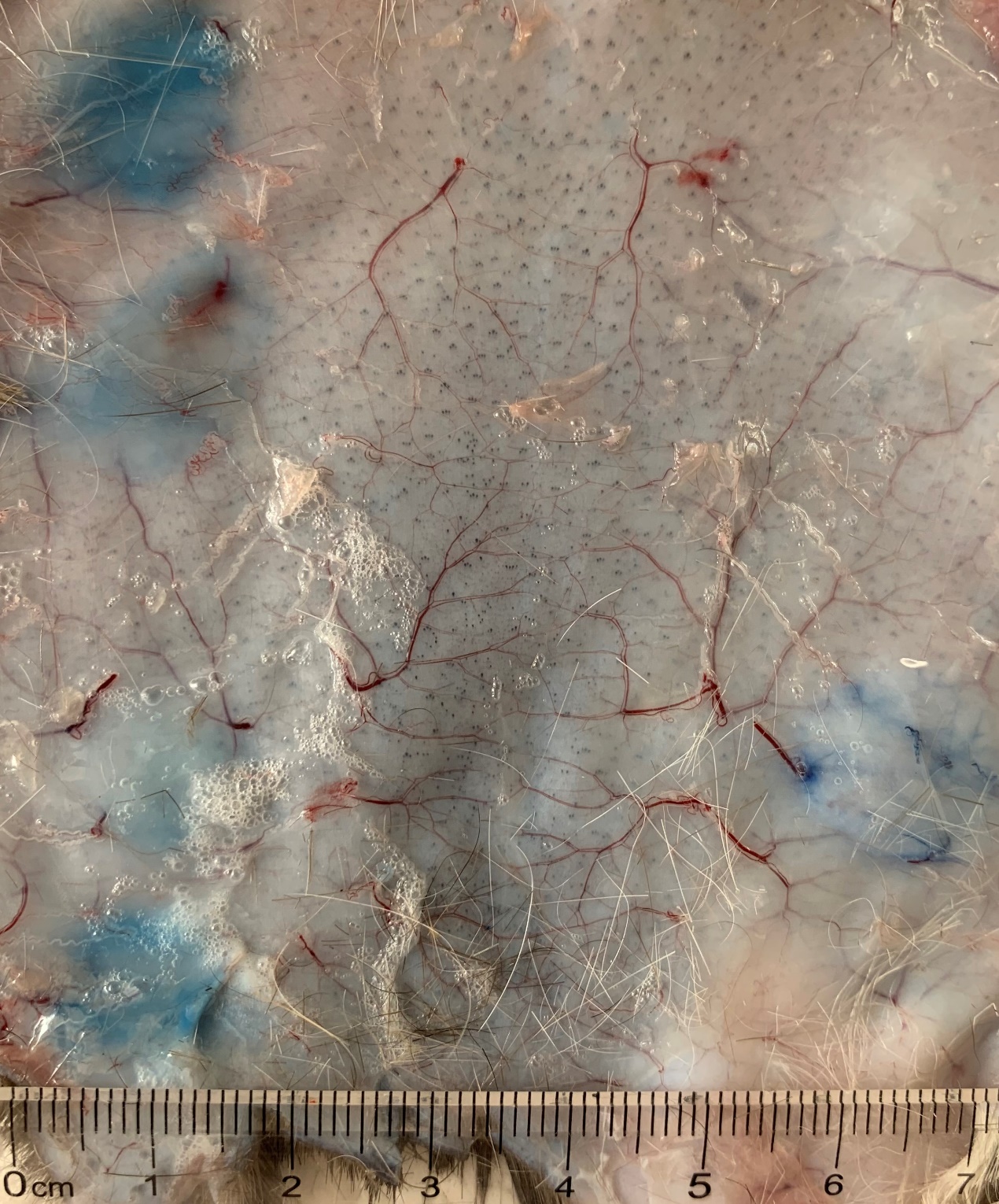

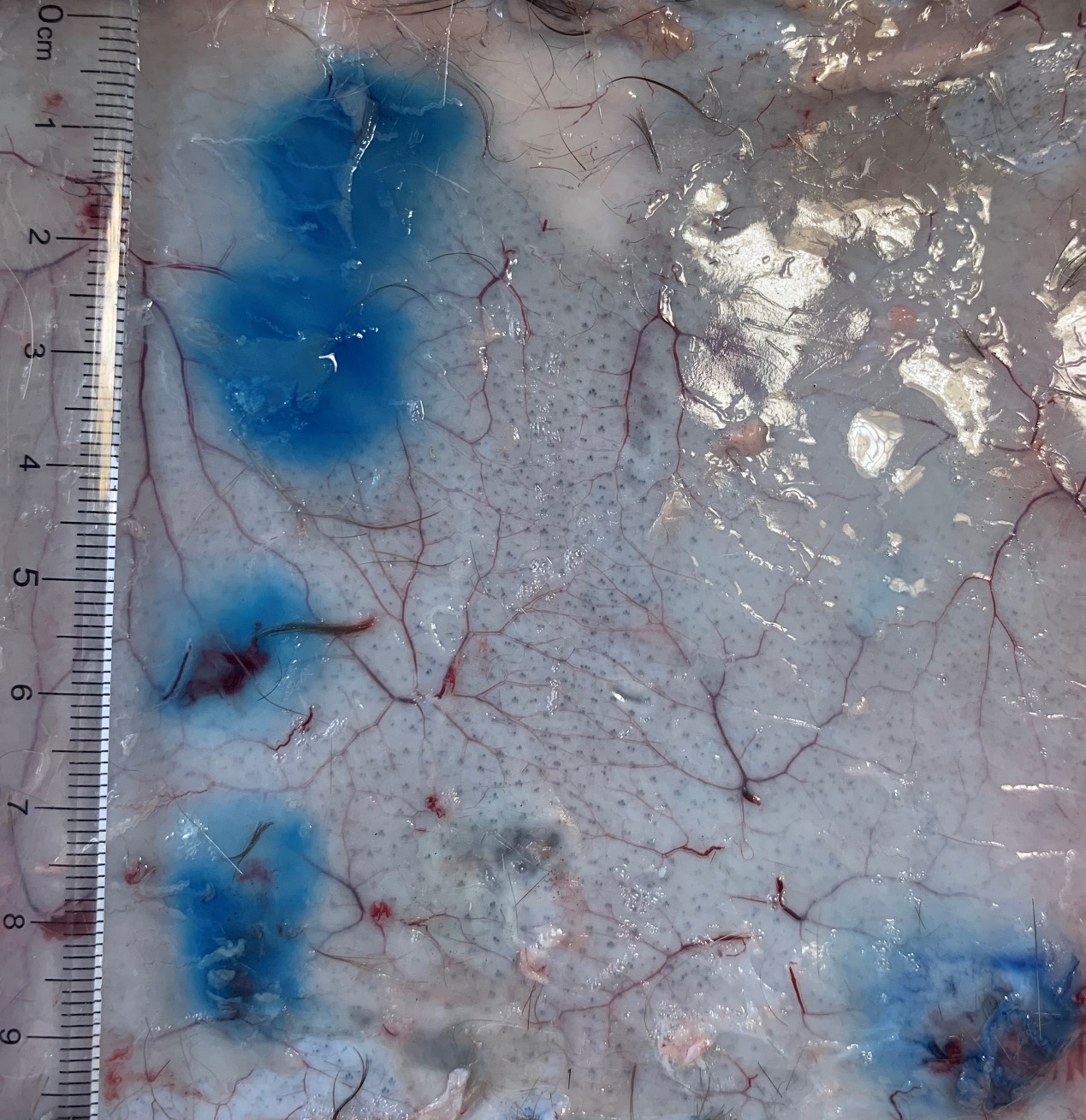

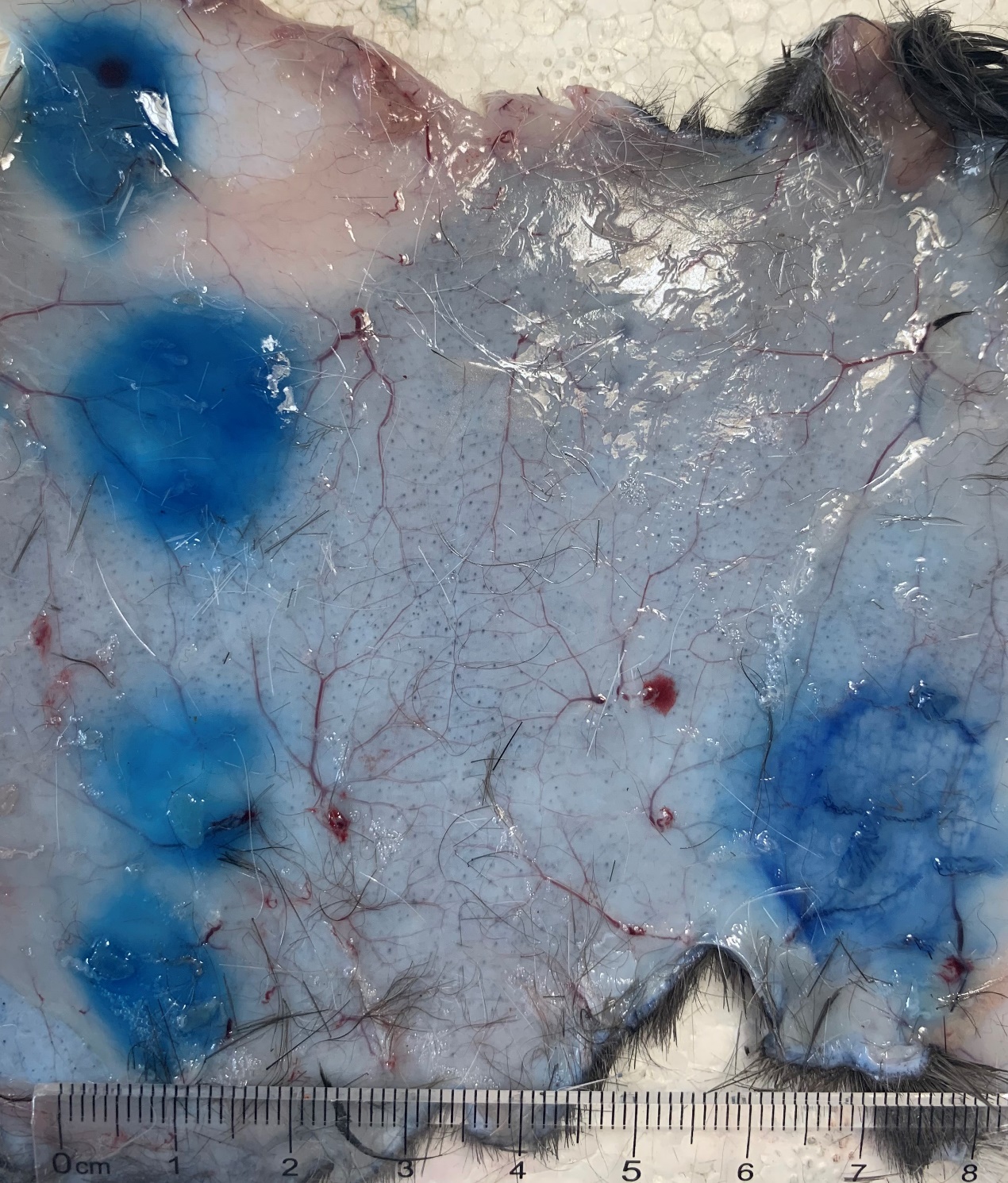

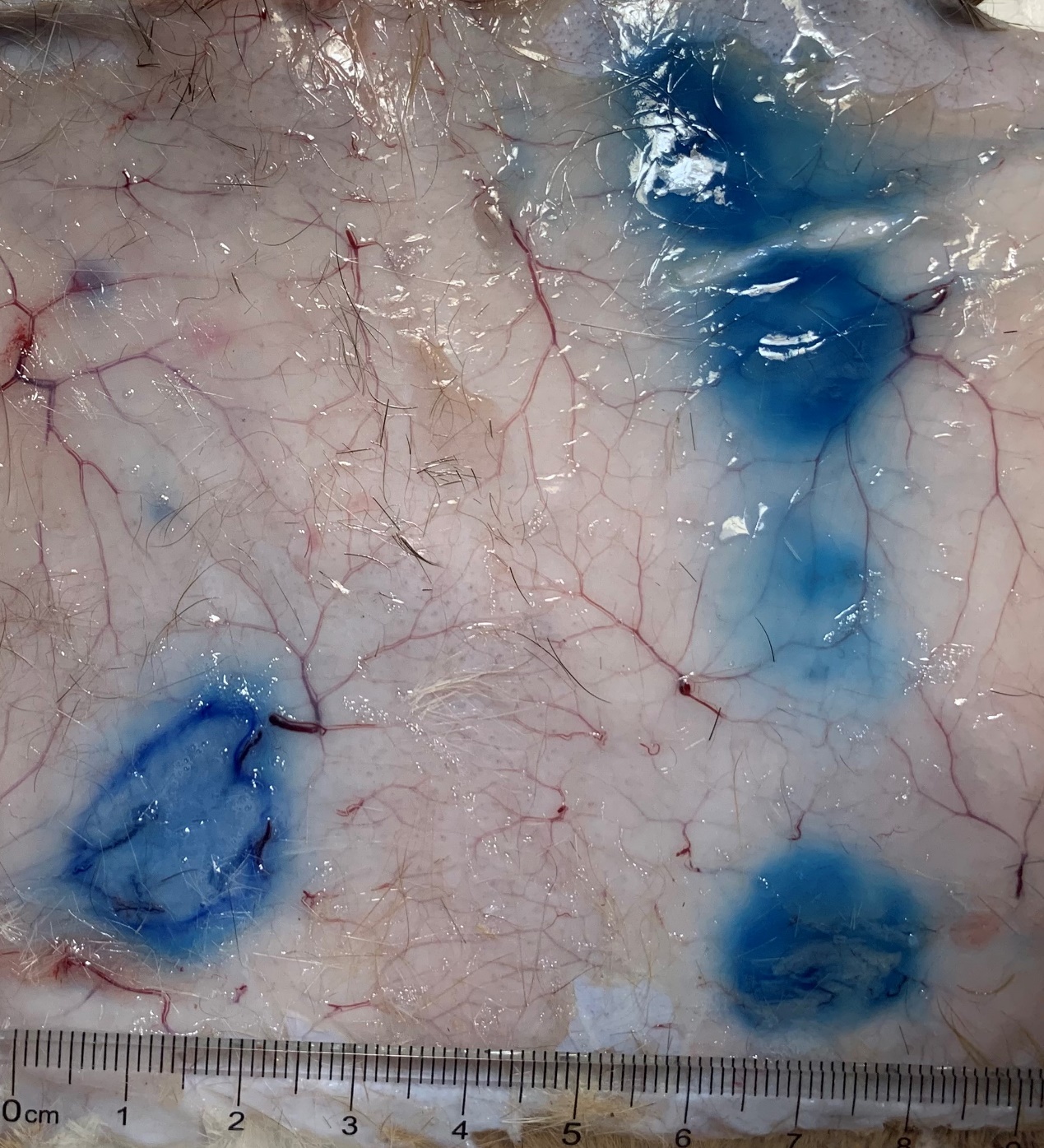


## 6.2 Raw data for 6B


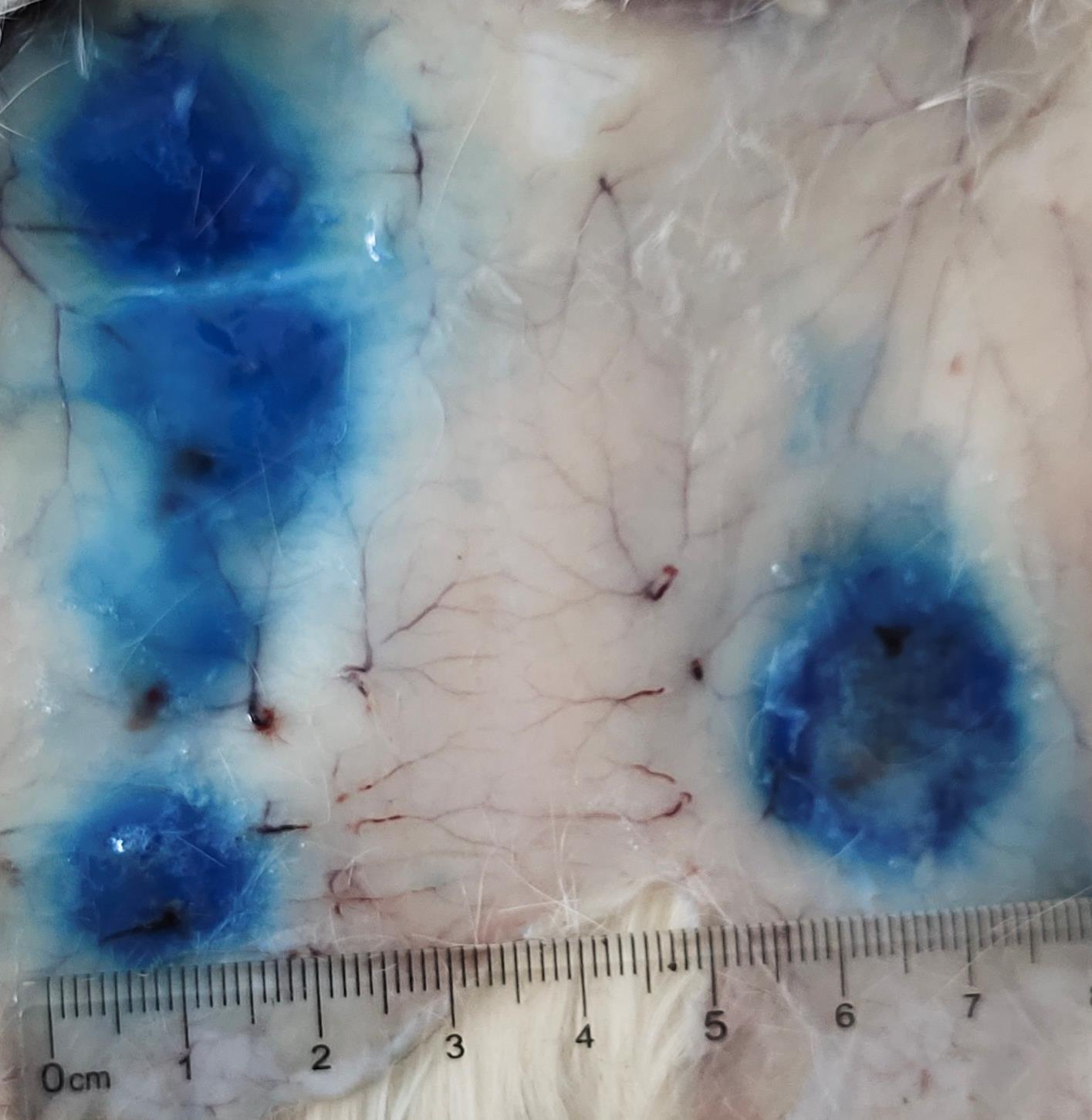

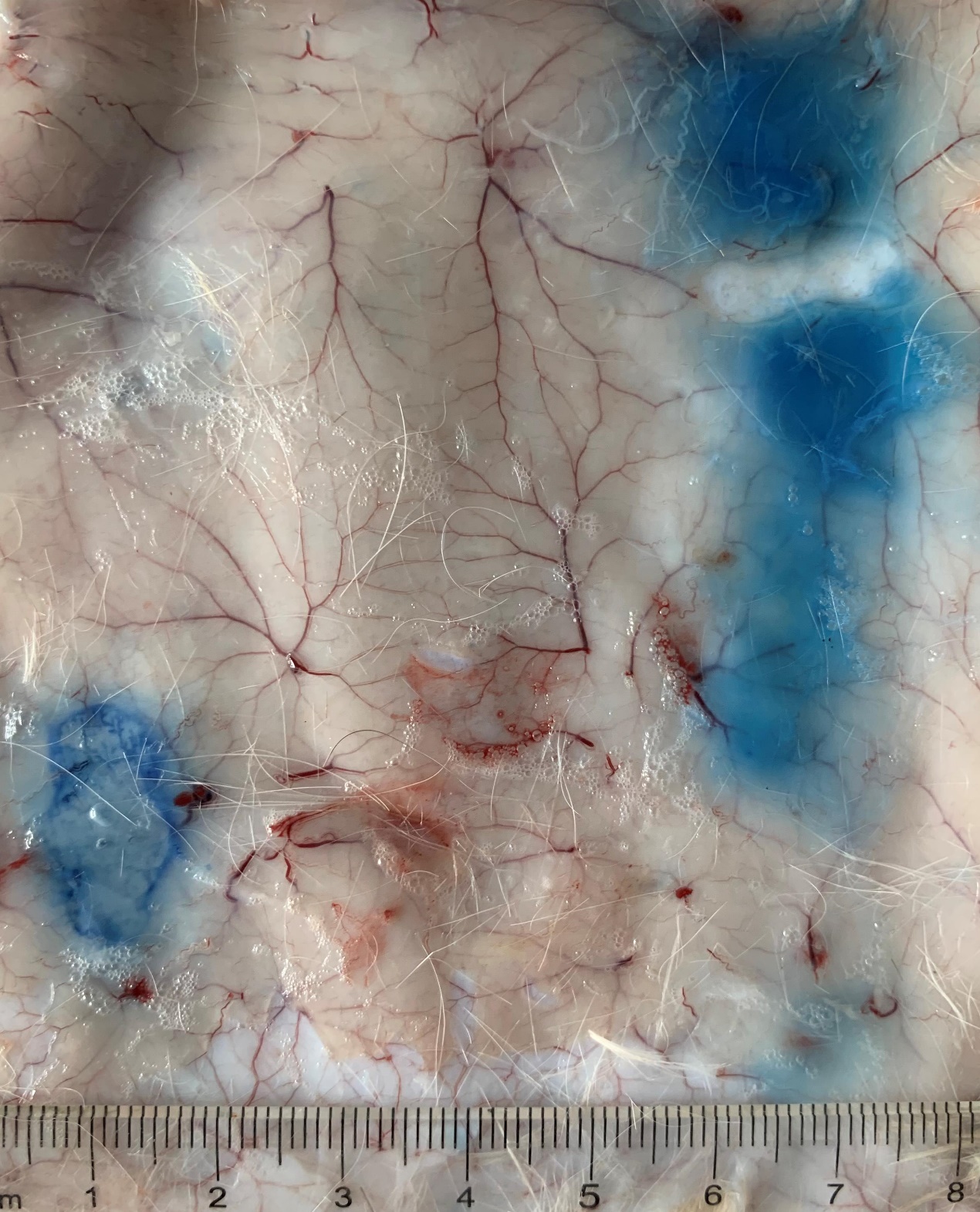

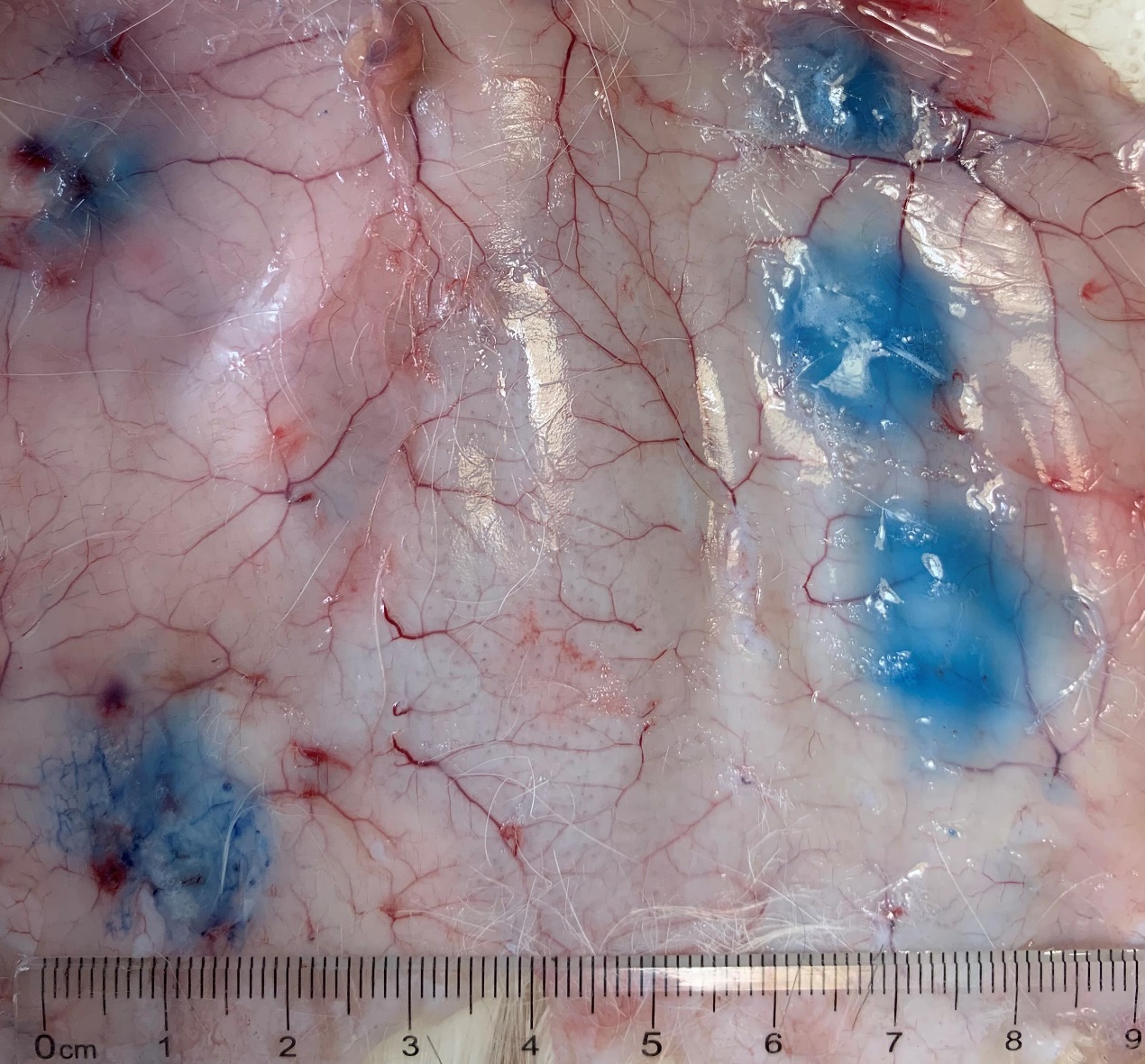

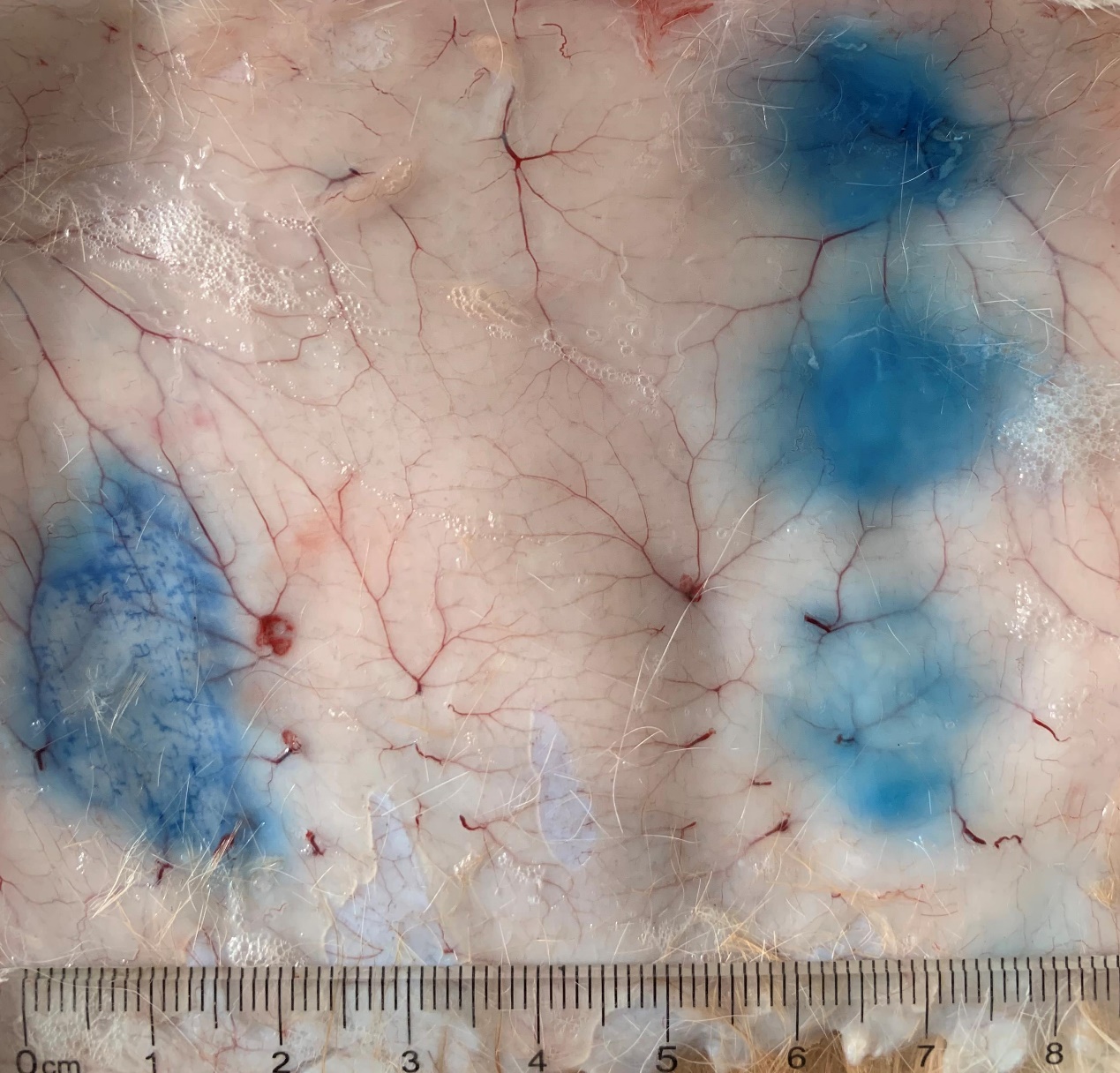


## 6.3 Raw data for Fig 6C without cromolyn sodium (CS): from 6.1

| ug/cm2 | 1 | 2 | 3 | 4 | Mean | SD | P1vs NS | P2 Vs Ori |
| --- | --- | --- | --- | --- | --- | --- | --- | --- |
| Com A | 0.472 | 0.519 | 0.396 | 0.416 | 0.451 | 0.056 | 0.000 | 0.114 |
| ComB | 0.484 | 0.423 | 0.378 | 0.439 | 0.431 | 0.044 | 0.000 | 0.189 |
| Com C | 0.281 | 0.162 | 0.187 | 0.248 | 0.220 | 0.055 | 0.175 | 0.006 |
| Ori | 0.437 | 0.320 | 0.412 | 0.345 | 0.379 | 0.055 | 0.001 | 1.000 |
| NS | 0.160 | 0.162 | 0.115 | 0.224 | 0.166 | 0.045 | 1.000 | 0.001 |
| C48/80 | 0.474 | 0.357 | 0.339 | 0.283 | 0.363 | 0.080 | 0.005 | 0.763 |

## 6.4 Raw data for Fig 6C with cromolyn sodium (CS) (50 mg/kg) : from 6.2

| ug/cm2 | 1 | 2 | 3 | 4 | Mean | SD | P1vs NS | P2 Vs Ori |
| --- | --- | --- | --- | --- | --- | --- | --- | --- |
| Com A | 0.285 | 0.313 | 0.142 | 0.345 | 0.271 | 0.090 | 0.003 | 0.068 |
| ComB | 0.203 | 0.115 | 0.202 | 0.149 | 0.168 | 0.043 | 0.003 | 0.942 |
| Com C | 0.087 | 0.034 | 0.081 | 0.046 | 0.062 | 0.026 | 0.340 | 0.002 |
| Ori | 0.212 | 0.154 | 0.160 | 0.136 | 0.166 | 0.032 | 0.002 | 1.000 |
| NS | 0.037 | 0.013 | 0.021 | 0.088 | 0.040 | 0.034 | 1.000 | 0.002 |
| C48/80 | 0.261 | 0.262 | 0.198 | 0.117 | 0.210 | 0.068 | 0.004 | 0.289 |

# 7 Raw data for Figure 10

| Concentration(mg/ml) | Com A | Com B | Com C | original |
| --- | --- | --- | --- | --- |
| 6 | 100 | 100 | 0 | 100 |
| 3 | 100 | 100 | 0 | 100 |
| 1.5 | 95.6 | 95.9 | 0 | 88.4 |
| 0.75 | 94.6 | 88.2 | 0 | 80.4 |
| 0.375 | 72.5 | 57.3 | 0 | 50.6 |
| 0.1875 | 45.5 | 30.4 | 0 | 25.8 |
| 0.09375 | 22.4 | 16.5 | 0 | 12.5 |
| 0.046875 | 6 | 3.3 | 0 | 1.1 |
